# Nanogel Integrated Zwitterionic Injectable Hydrogel with Sequential Drug‐Releasing Capability for the Programmable Repair of Spinal Cord Injury

**DOI:** 10.1002/advs.202510976

**Published:** 2025-11-12

**Authors:** Zhijian Wei, Susu Huang, Wencan Zhang, Jiayao Wen, Xiaolong Zhou, Wei He, Xiaoye Yang, Haifeng Wang, Guangxi Zhai, Bin Shi, Lin Jin, Dachuan Wang, Shiqing Feng, Lei Ye

**Affiliations:** ^1^ NMPA Key Laboratory for Technology Research and Evaluation of Drug Products and Key Laboratory of Chemical Biology (Ministry of Education) State Key Laboratory of Discovery and Utilization of Functional Components in Traditional Chinese Medicine Department of Pharmaceutics School of Pharmaceutical Sciences Cheeloo College of Medicine Shandong University No. 44 Wenhua West Road, Lixia District Jinan 250012 China; ^2^ Department of Orthopaedics Qilu Hospital, Shandong University Centre for Orthopaedics Advanced Medical Research Institute Shandong University Jinan Shandong 250012 China; ^3^ Department of Orthopaedics The Second Hospital of Shandong University No. 247 Beiyuan Street, Tianqiao District Jinan 250033 China; ^4^ Department of Pharmacy Zhongshan Hospital Fudan University 180 Fenglin Road Shanghai 200032 China; ^5^ International Joint Research Laboratory for Biomedical Nanomaterials of Henan Zhoukou Normal University No. 6, Middle Section of Wenchang Avenue, Chuanhui District Zhoukou 466001 China; ^6^ Neck‐Shoulder and Lumbocrural Pain Hospital of Shandong First Medical University Shandong First Medical University & Shandong Academy of Medical Sciences No. 18877, Jingshi Road, Lixia District Jinan 518873 China

**Keywords:** hydrogel, injectable, melatonin, spinal cord injury, zwitterionic

## Abstract

Spinal cord injury (SCI), a highly disabling injury to the central nervous system, has a complex and sequential pathogenesis. Traditional multiple‐delivery systems rely on a physical mix of nanoparticles or drugs in hydrogels, which lacks the controllability of the drug. To solve these problems, an integrative hydrogel system cross‐linked with a Per‐g‐PSB nanogel is designed. Dendritic macromolecular nanogels (Per‐g‐PSB) could not only act as cross‐linkers to adjust the hydrogel mechanical properties but also form a dynamic network (Dex/Per‐g‐PSB hydrogel). Most importantly, a large amount of charge could lead to a significant sustained release via electrostatic interactions. The hydrogel platform (Mel/Ibu@D/P‐g‐PSB) realizes the sequential release of melatonin and ibuprofen by different mechanisms. Melatonin is first released by diffusion and exhibits significant neuroprotective effects during the acute phase by downregulating the expression of inflammatory cytokines. Ibuprofen is released in the second stage due to strong electrostatic interactions, and it reduces RhoA signaling activation by blocking the ROCK pathway in the subacute phase, which reduces the deleterious cascade reaction after spinal cord injury. These results show that the ion‐sensitive hydrogel platform with sequentially releasing drug effects, combining anti‐inflammatory effects and blocking the ROCK pathway, shows excellent repair of SCI.

## Graphical abstract

A novel drug‐loaded D/P‐g‐PSB nanogel‐incorporated hydrogel by the electrostatic attraction‐driven self‐assembling process. Sequential drug releasing property (melatonin is released first by physical diffusion, and then ibuprofen is released as the charge shielding effect and hydrogel degradation). This ion‐sensitive hydrogel platform with sequentially releasing drug effects, combining anti‐inflammatory effects and blocking the ROCK pathway, shows excellent repair of SCI.

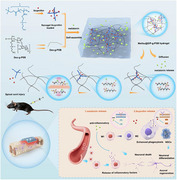

## 1 Introduction

Spinal cord injury (SCI) is a highly disabling injury to the central nervous system (CNS) that triggers sequelae, such as permanent loss of sensory and motor functions or even death.^[^ ^1^, ^2^, ^3^ ^]^ SCI is immediately followed by infiltration of spinal cord tissue with inflammatory factors.^[^ ^4^ ^]^ Subsequently, astrocyte activation occurs, preventing axonal regeneration and leading to permanent damage.^[^ ^5^, ^6^, ^7^ ^]^ Drug therapy,^[^ ^8^, ^9^, ^10^ ^]^ cell transplantation,^[^ ^11^, ^12^ ^]^ and bioscaffolds^[^ ^13^, ^14^ ^]^ have been extensively studied for the repair of SCI.^[^ ^15^, ^16^ ^]^

Several strategies have been proposed based on the complex pathology and recovery stages of SCI. However, current therapies focus on a single target and fail to account for the dynamic changes in the microenvironment at the lesion site, which impedes recovery from SCI. Dai et al.^[^ ^17^ ^]^ prepared an MMP‐responsive GCH hydrogel loaded with recombinant protein GST‐TIMP‐bFGF to inhibit MMP levels and promote the recovery of motor function after spinal cord injury. Liu et al.^[^ ^16^ ^]^ prepared a CS‐CA‐DA hydrogel with excellent mechanical properties that exhibited good cell adhesion ability and promoted cell and axonal regeneration in the damaged area. However, these gels only promoted the repair of SCI in a single target and were unable to cover the sequential treatment process to match different SCI microenvironments. Although single‐target‐based therapy has proven its efficacy in animal models, its effects are limited, hindering its translation to clinical applications. Most importantly, in conventional injectable hydrogels, nanoparticles are loaded by random blending. There are no interactions between the nanoparticles and the hydrogel skeleton, and the release mechanism is simple diffusion, which cannot precisely control drug release. Therefore, there is an urgent need to develop a versatile and more effective integrated hydrogel system with “real” sequential drug‐releasing properties for dynamic treatment.

Pentaerythritol (Per) is a polyol containing four equivalent methyl groups with a high degree of symmetry, whose side chains could be acylated to prepare star polymers with a pentaerythritol core.^[^ ^18^, ^19^ ^]^ A four‐armed polymer with zwitterionic chain segments could self‐assemble into a nanogel via electrostatic interactions. Model drugs with negative or positive charges could couple with the zwitterionic hydrogel backbone, leading to sequential sustained drug release properties such as hydrogel degradation.^[^ ^20^, ^21^, ^22^, ^23^ ^]^ This smart drug delivery platform could perfectly match the dynamic pathological changes of SCI.

The most important pathological change in SCI is severe inflammation around the injured area, which starts just a few minutes after the injury and may persist for months.^[^ ^10^, ^24^ ^]^ Inflammation after SCI is complex and driven by a diverse set of cells and inflammatory cytokines.^[^ ^25^ ^]^ Microglia, the CNS resident macrophages, quickly respond to injury by changing into a pro‐inflammatory phenotype and releasing cytokines, which leads to the infiltration of peripheral immune cells, especially bone marrow‐derived macrophages.^[^ ^26^ ^]^ In injured conditions, CNS macrophages exhibit different phenotypes and functions, such as pro‐inflammatory macrophages (M1) and anti‐inflammatory macrophages (M2). The M1 macrophages increase the expression of inflammatory cytokines, such as TNF‐α, IL‐1β, and IL‐12, aggravate tissue damage, and hinder injury repair. By contrast, M2 macrophages remove necrotic tissue debris and release protective factors, including IL‐4 and IL‐10.^[^ ^27^ ^]^ At an early stage after injury, most microglia/macrophages present the M1 phenotype, with only a few M2 polarization cells. Melatonin is an indole hormone that inhibits the inflammatory response and lowers IL‐1β levels.^[^ ^28^ ^]^ It was reported to reduce inflammation, promote microglia M2 polarization, and reduce apoptosis during the acute phase of spinal cord injury in mice.^[^ ^29^, ^30^ ^]^

The inflammatory cascade reaction following spinal cord injury persists into the subacute phase. During this period, the RhoA/ROCK pathway is activated by neurons and glial cells.^[^ ^31^, ^32^ ^]^ The activation of RhoA was found to induce growth cone collapse, prevent axon elongation, and cause neuronal and glial cell apoptosis.^[^ ^33^, ^34^, ^35^ ^]^ This way, the inflammatory microenvironment was remolded. Subsequently, ibuprofen was released, which exerted its anti‐inflammatory effects as a nonsteroidal anti‐inflammatory drug and as a ROCK pathway inhibitor to promote neuronal differentiation.^[^ ^33^ ^]^ Phagocytosis is an important function of microglia/macrophages for removing debris and establishing a compatible microenvironment, and the RhoA/ROCK pathway is closely linked to phagocytosis of microglia/macrophages. Phagocytosis mediated by M1 phenotype was shown to aggravate tissue damage, whereas phagocytosis mediated by the M2 phenotype exhibited neuroprotective functions.^[^ ^36^ ^]^ Therefore, blocking ROCK pathway inhibitors to enhance the phagocytosis of microglia/macrophages at the subacute stage was deemed a good strategy for SCI repair. By matching different recovery periods, this smart drug delivery platform is an ideal strategy for spinal cord injury clinical treatment of SCI.

Based on the above analysis, we designed a Mel/Ibu@D/P‐g‐PSB hydrogel drug delivery system. Distinguishing from the conventional “nano‐hydrogel” composite drug delivery system, the PSB‐grafted nanogel Per‐g‐PSB has the same pendant of PSB as Dex‐g‐PSB, forming the integrated delivery system. The Per‐g‐PSB nanogel serves the dual roles of a cross‐linker and a drug carrier. As a cross‐linker, the four arms of the PSB segments react with the Dex‐g‐PSB hydrogel skeleton structure and adjust the mechanism of the hydrogel to match the spinal tissue for better compatibility and treatment. On the other hand, the PSB in Dex and Per exhibits strong electrostatic interactions between the nanogel and the hydrogel. Its dendritic structure with a large amount of charge makes it an ideal drug carrier for loading ibuprofen into its cavity via electrostatic interactions, leading to a significantly prolonged drug delivery. In this sequential drug delivery system, melatonin, released first by diffusion, exerts anti‐inflammatory effects, promotes microglial M2 polarization, and reduces apoptosis in the acute phase. As the hydrogel degrades and the P‐g‐PSB cross‐linker is released, ibuprofen is released from the cross‐linker. This results in a prolonged release period compared to melatonin and inhibits the ROCK pathway to promote phagocytosis of microglia/macrophages in the later stages of SCI. The in vitro and in vivo results demonstrated that this novel injectable hydrogel system with sequential drug‐releasing capability is effective in the programmable repair of SCI, providing great therapeutic potential for the clinical translation of SCI repair.

## 2 Results and Discussion

**Scheme 1:**
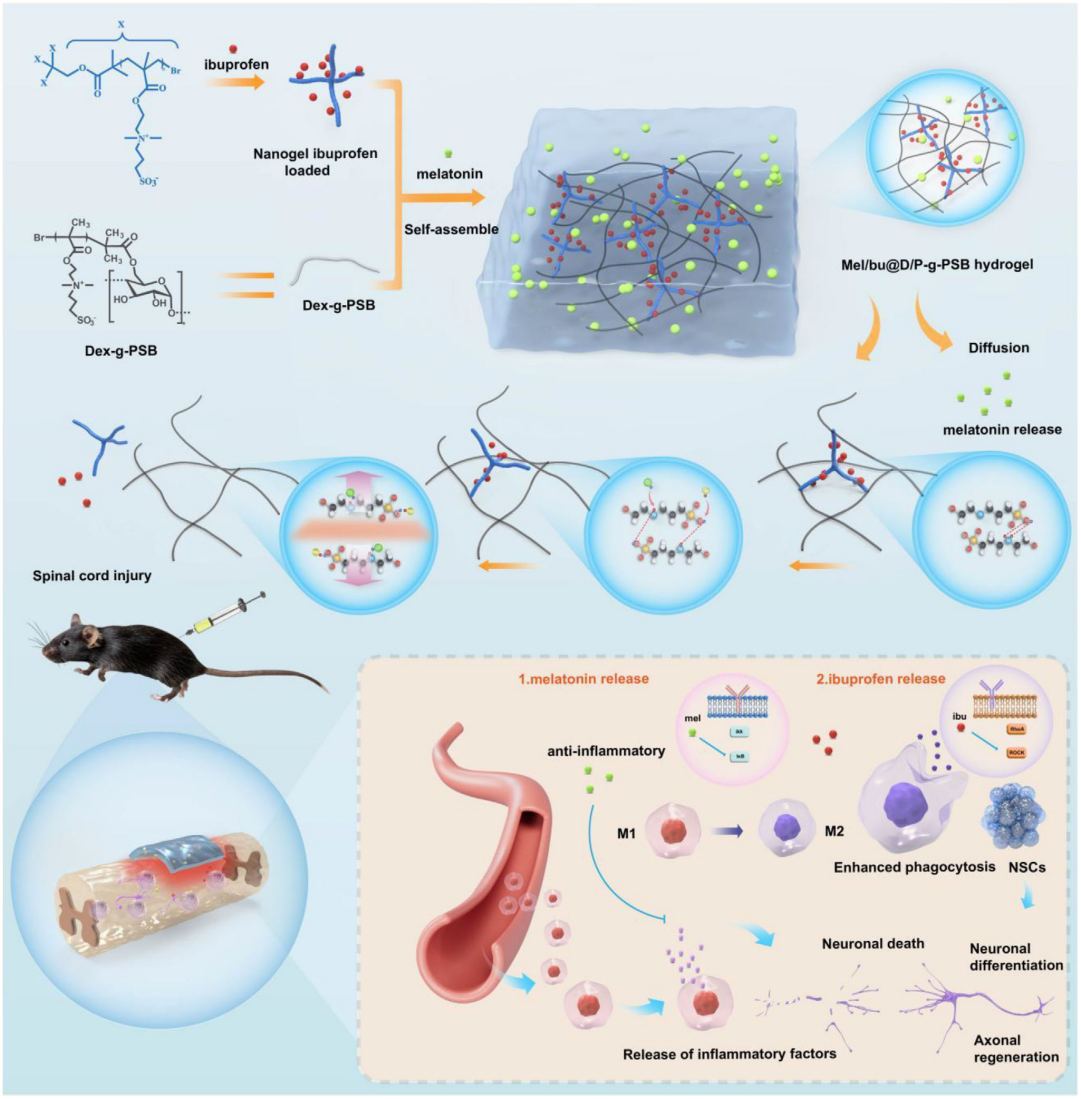
Schematic illustration of the nanogel‐incorporated hydrogel preparation process, sequential drug‐releasing, and potential mechanism. a) Preparation of drug‐loaded D/P‐g‐PSB hydrogel by the electrostatic attraction‐driven self‐assemble process; b) Sequential drug releasing property (melatonin released first by the physical diffusion, and then ibuprofen was released as the charge shielding effect and hydrogel degradation); c) Schematic of the potential molecular mechanism of Mel/Ibu@D/P‐g‐PSB hydrogel platform, which promoted the spinal cord regeneration.

A facile therapeutic system with a sequential release effect for the programmable repair of the spinal cord injury was designed by loading melatonin and ibuprofen into the zwitterionic physical hydrogel. By incorporating the ibuprofen‐loaded nanogel, the mechanical properties could be adjusted to match the spinal tissue for better injury recovery. These two drugs could be controlled for a sequential drug release. Melatonin was first released by physical diffusion, and then ibuprofen was released as the hydrogel degraded. The results of the in vitro and in vivo experiments confirmed that melatonin released first by diffusion exerted anti‐inflammatory effects, promoted macrophage M2 polarization, and reduced apoptosis in the early stage. Subsequently, ibuprofen was released for a longer time and inhibited the ROCK pathway to promote neuronal differentiation in the subsequent stages of spinal cord injury (**Scheme** **1**).

### 2.1 Synthesis and Characterization of Per‐Br, Per/Dex‐g‐PSB

The Dex‐g‐PSB copolymers were synthesized according to our laboratory protocols, as shown in Table S1 (Supporting Information). Because the yield began to decrease when the degree of polymerization was increased to 240, the Dex‐g‐PSB polymer with a degree of polymerization of 180 was selected for subsequent experiments.

Per‐Br was synthesized by the esterification of pentaerythritol with BIBB (Figure S1a, Supporting Information). The structure of the synthesized four‐armed initiator was characterized by ^1^H‐NMR, as shown in Figure S1c (Supporting Information), where the strong peak at a chemical shift value of 1.9 ppm belonged to the methyl proton peak (–CH(CH_3_)_3_) in BIBB. In addition, the proton peak located at 4.28 ppm was attributed to a methylene group (–CH_2_) located between the C and O atoms in the pentaerythritol structure. Based on the ratio of the areas of peaks 1 and 2, the degree of substitution of Per was determined to be 4. The structure of Per‐Br was also characterized using FT‐IR spectroscopy. Per had a strong and broad absorption band at 3300 cm^−1^ corresponding to the –OH stretching vibration of the hydroxyl group. The FT‐IR spectra of Per and Per‐Br were significantly different, with a strong adsorption peak attributed to –C═O stretching vibration at 1730 cm^−1^ for Per‐Br, indicating the presence of ester groups in Per‐Br (Figure S2, Supporting Information). These results indicated that Per‐Br was successfully synthesized by the esterification of pentaerythritol with BIBB.

The synthesis route for Per‐g‐PSB is shown in Figure S1c (Supporting Information). The four‐armed initiator Per‐Br was graft‐polymerized with the SBMA monomer by ATRP reaction to obtain Per‐g‐PSB (Figure S1b, Supporting Information). As shown in Figure S1d (Supporting Information), the structure of Per‐g‐PSB was characterized by ^1^H‐NMR. A hydrogen proton peak is shown in Figure S1d (Supporting Information). In Figure S2 (Supporting Information), the absorption peaks at 1020 and 1130 cm^−1^ in Per‐g‐PSB were mainly attributed to the symmetric and asymmetric stretching vibrations of the S═O group. The absorption peaks at 992 and 1470 cm^−1^ were mainly attributed to the stretching vibrations of the S═O group and the C–N group. These results indicated the successful grafting of SBMA with pentaerythritol. Then, we performed some experiments to characterize the Per‐g‐PSB nanogels. First, the particle size and zeta potential of the Per‐g‐PSB nanogels were tested using a Malvern particle size analyzer. The results (Figure S3, Supporting Information) showed that the average particle size of the Per‐g‐PSB nanogels was 176.4 nm and the Zeta potential was 13.2mV. Then, the TEM image (Figure S5, Supporting Information) revealed that Per‐g‐PSB nanogels were uniform spherical particles with an average size of ≈200 nm, which was similar to the result of the particle size analyzer. This spherical morphology is likely caused by the curling of the pSB chains grafted onto Per, driven by electrostatic interactions between the pSB segments.

**Figure 1:**
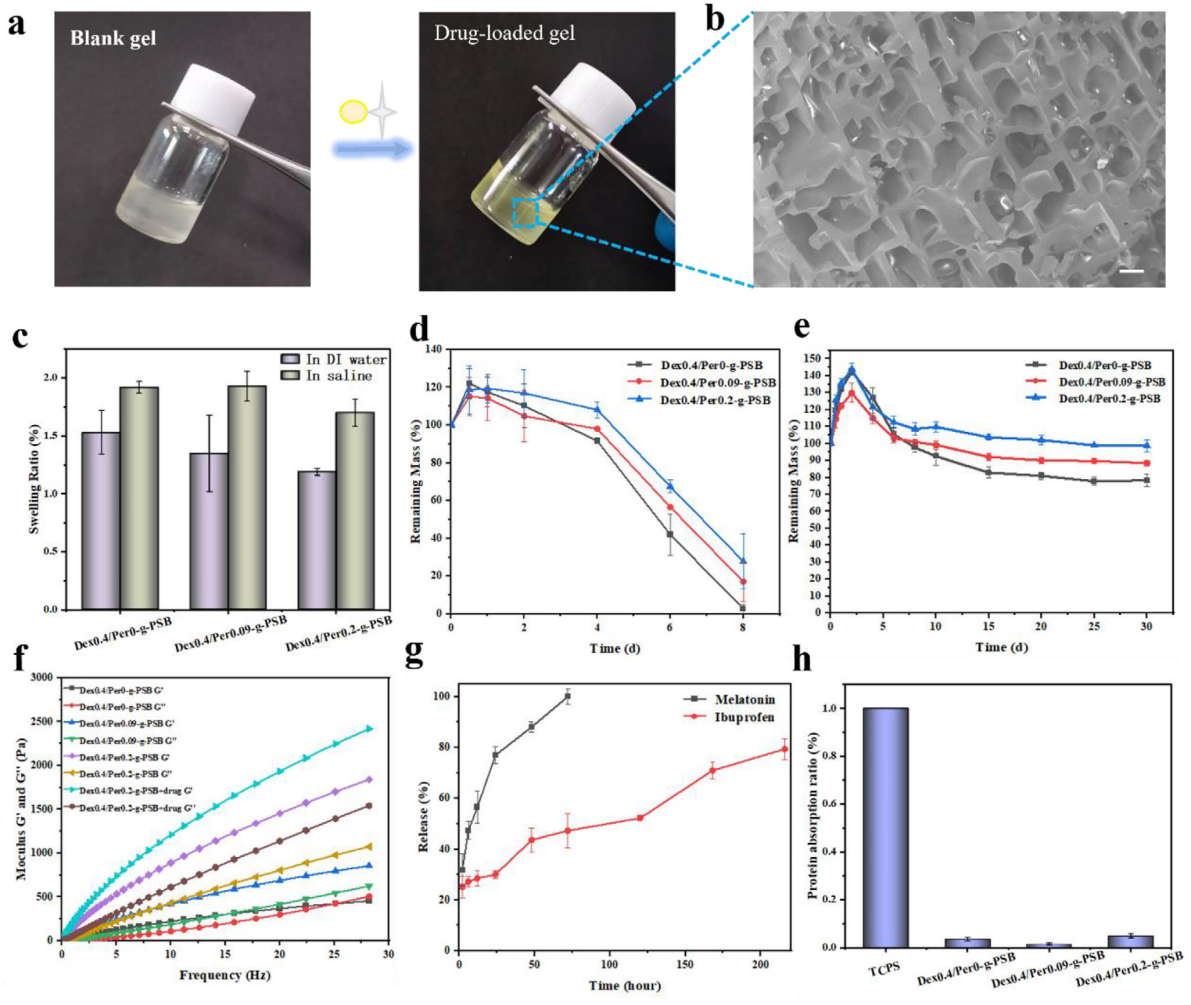
Characterization of D/P‐g‐PSB hydrogels. a) Digital photos of blank and drug‐loaded hydrogels; b) SEM morphology. Scale bar: 20 µm; c) Swelling ratios of D/P‐g‐PSB hydrogels in DI water or saline at 37 °C; degradation behaviors of D/P‐g‐PSB hydrogels in d) PBS and e) DI water media; f) Mechanical properties of Dex/Per‐g‐PSB hydrogel (Storage modulus G′ and loss modulus G″); g) Release profile of drug from different hydrogels in PBS; h) Protein adsorption assay of Dex/Per‐g‐PSB hydrogel.

To prepare the Dex‐g‐Per‐PSB blank hydrogel and drug‐loaded hydrogel, the powders described in 2.3 and 2.4 were placed in vials based on the formulation shown in Table S2 (Supporting Information). The obtained Dex_a_/Per_b_‐g‐PSB hydrogels had final solid contents of 40%, 45%, and 50% (a denotes the mass of Dex‐g‐PSB powder, b denotes the mass of Per‐g‐PSB powder). Melatonin and ibuprofen were dissolved in PBS to prepare drug‐loaded hydrogels for subsequent experiments (**Figure** **1**a).

### 2.2 Characterization of D/P‐g‐PSB Hydrogel

#### 2.2.1 SEM

SEM images of the lyophilized hydrogels (Figure 1b; Figure S6, Supporting Information) showed that different percentages of the hydrogel had a 3D porous structure. As the mass of the Per‐g‐PSB nanogel increased, its porosity decreased. On the other hand, the network structure of the hydrogel could accommodate the nutrients required for cell proliferation.

#### 2.2.2 Swelling Ratio

The swelling rates of D/P‐g‐PSB hydrogels (Figure 1c) were lower in water than in saline. The charge‐shielding effect in saline was stronger owing to the presence of large amounts of sodium and chloride ions, which disrupted the cross‐linking points of the gel backbone. In addition, owing to the introduction of the Per‐g‐PSB nanogel, it acted as a physical cross‐linker to adjust the tissue‐matched hydrogel. On the other hand, the increased number of cross‐linking points in the hydrogel led to a lower swelling ratio.

#### 2.2.3 Rheological Test

The rheological test results (Figure 1f) showed that the mechanical strength of the hydrogel was as follows: 40% Dex/Per‐g‐PSB hydrogel < 45% Dex/Per‐g‐PSB hydrogel < 50% Dex/Per‐g‐PSB hydrogel. The G′ value increased gradually with the addition of the Per‐g‐PSB nanogel. In addition, the G′ values of the ibuprofen‐loaded hydrogel group were slightly higher than those of the unloaded group (50% Dex/Per‐g‐PSB hydrogel < 50% Ibu@Dex/Per‐g‐PSB hydrogel), further verifying the existence of electrostatic forces between Per‐g‐PSB nanogel and ibuprofen. This property is conducive to sequential drug release from hydrogels to achieve the best therapeutic effect. It is generally believed that the modulus of spinal cord tissue is between 0.1 and 3 kPa. According to the rheological test results, the storage modulus of Dex/Per‐g‐PSB hydrogels was adjustable between 0.2 and 2 kPa by controlling the content of Per‐g‐PSB nanogels, which is matchable with native spinal cord tissue.

#### 2.2.4 Drug Release In vitro and In vivo

Melatonin was physically added to and dispersed in the porous structure of the Dex‐g‐PSBMA hydrogel. The charged ibuprofen exhibited electrostatic interaction forces with the Per‐g‐PSB nanogel, which were linked and trapped in the nanogel cavity. In PBS, physically mixed melatonin was first released from the Dex‐g‐PSB hydrogel backbone by diffusion, as shown in Figure 1g. Thereafter, with the gradual infiltration of ions, the Dex/Per‐g‐PSB hydrogel skeleton was subjected to charge shielding, and the cross‐linking points between the Per‐g‐PSBMA microgels and ibuprofen were disrupted. Subsequently, ibuprofen was gradually released from the Per‐g‐PSB nanogels. The entire drug release stage lasted for ≈14 days, and the addition of the nanogel reinforced the gel skeleton and slowed its disintegration.

The in vitro drug release of the Mel/Ibu@D/P‐g‐PSB hydrogel was determined by high‐performance liquid chromatography (HPLC). The HPLC results show that the retention time of the blank plasma is ≈3 min (Figure S7a, Supporting Information), the retention time of melatonin in plasma containing melatonin is 4.5 min (Figure S7b, Supporting Information), and the retention time of ibuprofen in plasma containing ibuprofen is 6 min (Figure S7c, Supporting Information), with all the resolutions greater than 1.5, which meets the testing requirements. Ultimately, the HPLC results of the medicated plasma indicated that the testing method is effective. The blood concentration‐time curve of ibuprofen and melatonin (Figure S8, Supporting Information) showed that the blood concentration of melatonin was always higher than that of ibuprofen at the same sampling time, indicating that melatonin is released before ibuprofen. This further confirms our idea: melatonin rapidly enters the bloodstream due to diffusion, while ibuprofen is released as the gel slowly degrades, because it binds to the gel matrix through electrostatic interactions.

#### 2.2.5 Degradation Rate

The degradation behavior of D/P‐g‐PSB hydrogels was evaluated in water and PBS. As shown in Figure 1d, the D/P‐g‐PSB hydrogel in PBS began to degrade after undergoing a swelling process and reached swelling equilibrium. With charge infiltration, hydrogel degradation accelerated, and the degradation was complete in ≈8 days. After the addition of the nanogel cross‐linker Per‐g‐PSB, degradation of the Dex/Per‐g‐PSB hydrogel was significantly slower than that of the uncross‐linked group. The introduction of the Per‐g‐PSB nanogel strengthened the gel skeleton, and as the number of cross‐linking points increased under the same number of ions, gel degradation slowed. In addition, the Dex/Per‐g‐PSB hydrogel degraded ≈25% (Figure 1e) over a period of 30 days in DI water. The degradation rate in DI water was much lower than that in PBS. The in vivo degradation results of Mel/Ibu@D/P‐g‐PSB hydrogel (Figure S9, Supporting Information) showed that the fluorescence of the hydrogel at the injury site could be maintained for up to 18 days, which is attributed to the good mechanical strength of the hydrogel. In addition, the slow in vivo degradation also provides a basis for sustained drug release.

#### 2.2.6 Hemolysis Analysis

The Dex/Per‐g‐PSB hydrogel was in direct contact with the trauma when used as a therapeutic carrier for spinal cord injury. After co‐incubating the gel extract with 2% erythrocyte suspension, the hemolysis rates were all lower than 5% (Figure S10, Supporting Information), indicating its good blood compatibility.

#### 2.2.7 Anti‐Adhesion Effect

In the treatment of spinal cord injuries, postoperative adhesions of the dural sac and nerve roots to the surrounding tissues are one of the most important causes of undesired therapeutic effects. The hydrogel could first act as a physical barrier to isolate damaged spinal cord tissue from the dura mater and adjacent nerve roots to avoid adhesions. In addition, Fibrous scarring formed by fibroblasts and the deposited laminin and fibronectin have a strong inhibitory effect on axonal regeneration. Zwitterionic polymers have a hydration effect and form a tight hydration layer on the membrane surface, effectively preventing contact between the proteins and the membrane surface, thereby inhibiting the overgrowth of scar tissue and providing enough space for axon extension and growth. As shown in Figure 1h, the adsorption of BSA on D/P‐g‐PSB hydrogel was 3.5%, 1.55%, and 4.85%, respectively, with TCPS as the control (100%), which exhibited excellent resistance to nonspecific protein adhesion.

**Figure 2:**
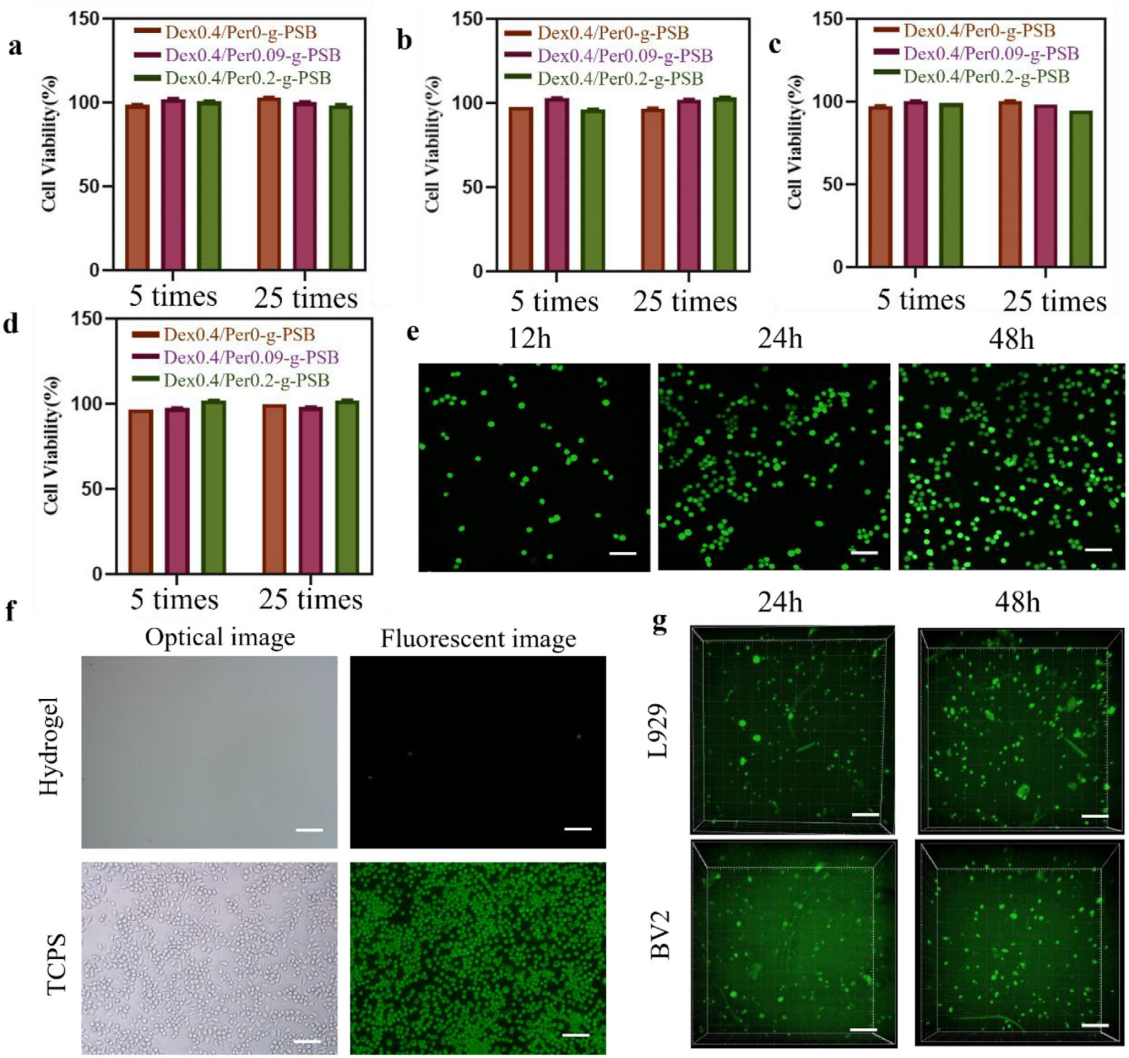
In vitro cytotoxicity assay of D/P‐g‐PSB hydrogel. Cell viability of hydrogel extracts against L929 cells for 12 h a) and 24 h b) incubation. Cell viability of hydrogel extracts against BV2 cells for 12 h c) and 24 h d) incubation; e) Live/dead staining images of BV2 cells at different time periods with a scale bar of 70 µm; f) Optical and stained images of cells on the substrates of D/P‐g‐PSB hydrogel and TCPS with a scale bar of 50 µm. g) 3D culture of L929 cells and BV2 cells in D/P‐g‐PSB hydrogel. Scale bar: 100 µm.

To further validate its anti‐adhesion properties, we used L929 cells to evaluate the ability of the Dex/Per‐g‐PSB hydrogel to resist cell adhesion. TCPS was used as a control group. The D/P‐g‐PSB hydrogel was seeded in 24‐well plates, and the cell suspension was added to the cells and co‐incubated for 24 h. The results (**Figure** **2**f) showed that L929 cells in the TCPS group were in an adherent growth state, and only a few sporadic cells were observed on the D/P‐g‐PSB hydrogel. The hydrated layer effectively prevented the adhesion of secreted proteins to the substrate surface and reduced cell adhesion, thus showing good resistance to nonspecific cell adhesion.

### 2.3 In vitro Cytotoxicity Assay

CCK‐8 results (Figure 2a–d) showed that there was no obvious cytotoxicity after co‐culturing with L929 cells and BV2 cells with different volumes of hydrogel extracts for 12 and 24 h, respectively, and the cell viability values were above 95%. In addition, live/dead staining showed similar results (Figure 2e), which did not show significant cytotoxicity after 12 h of incubation. After 24 and 48 h of co‐culture, the number of green‐stained cells increased, indicating that the cells began to proliferate.

In addition to the 2D culture, we verified cytotoxicity by co‐culturing cells with the hydrogels in a 3D culture. The cells encapsulated in the gel were stained after 24 and 48 h of incubation. Live/dead staining (Figure 2g) revealed a small number of cells (green fluorescence) after 24 h of incubation. After 48 h of co‐culture, the number of cells increased significantly, and clusters of cells appeared, suggesting that the cells grew well in the hydrogel. These results indicated that the Dex/Per‐g‐PSB hydrogel had excellent cytocompatibility.

### 2.4 In vitro Anti‐Inflammatory

**Figure 3:**
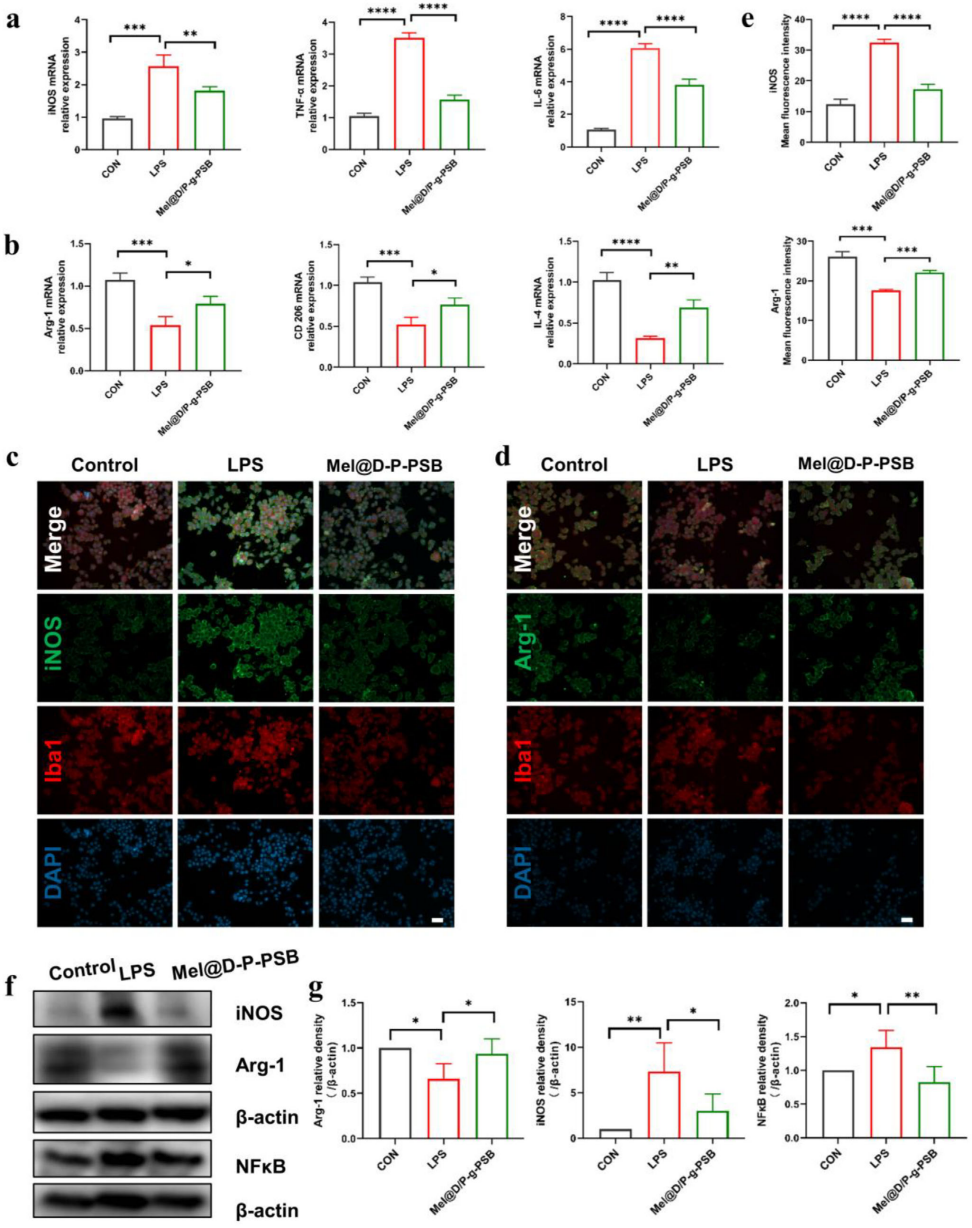
In vitro anti‐inflammatory effect of Mel @D/P‐g‐PSB hydrogel. PCR detection of hydrogel slow‐release melatonin Mel inhibits microglia, a) pro‐inflammatory M1‐related gene expression (*n* = 3) and b) anti‐inflammatory M2‐related gene expression (*n* = 3). Inhibition of microglia by melatonin was detected by fluorescence staining c) fluorescence intensity of pro‐inflammatory M1 marker iNOS; d) fluorescence intensity of anti‐inflammatory M2 marker Arg‐1. Scale bar: 50 µm; and e) fluorescence quantification results (*n* = 3). f) Western blot detection of melatonin inhibition of microglia pro‐inflammatory M1 marker iNOS protein expression and anti‐inflammatory M2 marker Arg‐1 protein expression; and g) protein quantification (*n* = 3). ^*^ *p* < 0.05, ^**^ *p* < 0.01, ^***^ *p* < 0.001, ^****^ *p* < 0.0001.

To validate the anti‐inflammatory effect of the Mel@D/P‐g‐PSB hydrogel in the acute phase of SCI, an in vitro simulated inflammatory environment was established using a Transwell chamber. A BV2 inflammation model was established, and the LPS induced morphological changes in BV2 cells—from round or round‐like to amoeboid or rod‐like morphology (Figure S11, Supporting Information) —which were inhibited by Mel@D/P‐g‐PSB treatment. The expression of pro‐inflammatory and anti‐inflammatory factors in BV2 cells before and after treatment with the Mel@D/P‐g‐PSB hydrogel was detected by PCR. The results, as shown in **Figure** **3**a,b, showed that compared with the LPS group, the TNF‐α and IL‐6 expressions decreased, and IL‐4 expression increased after treatment with Mel@D/P‐g‐PSB hydrogel. In contrast, iNOS mRNA expression was significantly lower, and Arg‐1 and CD206 mRNA expression were significantly higher in the Mel@D/P‐g‐PSB hydrogel group than in the LPS model group. This indicates that the Mel@D/P‐g‐PSB hydrogel treatment inhibited LPS‐induced neuroinflammatory responses in BV2 cells. Immunofluorescence staining results (Figure 3c–e) showed that after Mel@D/P‐g‐PSB hydrogel co‐incubation, Arg‐1 expression in BV2 cells was upregulated and iNOS expression was downregulated compared to that in the LPS group. The above results showed that the Mel@D/P‐g‐PSB hydrogel could induce the polarization of microglia from the M1 (pro‐inflammatory) to the M2 phenotype (anti‐inflammatory) in the acute phase.

The western blotting analysis and their quantitative results (Figure 3f,g) showed that compared with the LPS group, the expression of Arg‐1 protein was upregulated, the expression of iNOS protein was downregulated, and the expression of NF‐κB was significantly downregulated in BV2 cells after Mel@D/P‐g‐PSB hydrogel treatment. The above results suggest that melatonin could promote microglia polarization toward anti‐inflammatory type M2, a process associated with the inhibition of the NF‐κB pathway.

### 2.5 Inhibition of the ROCK Pathway In vitro

**Figure 4:**
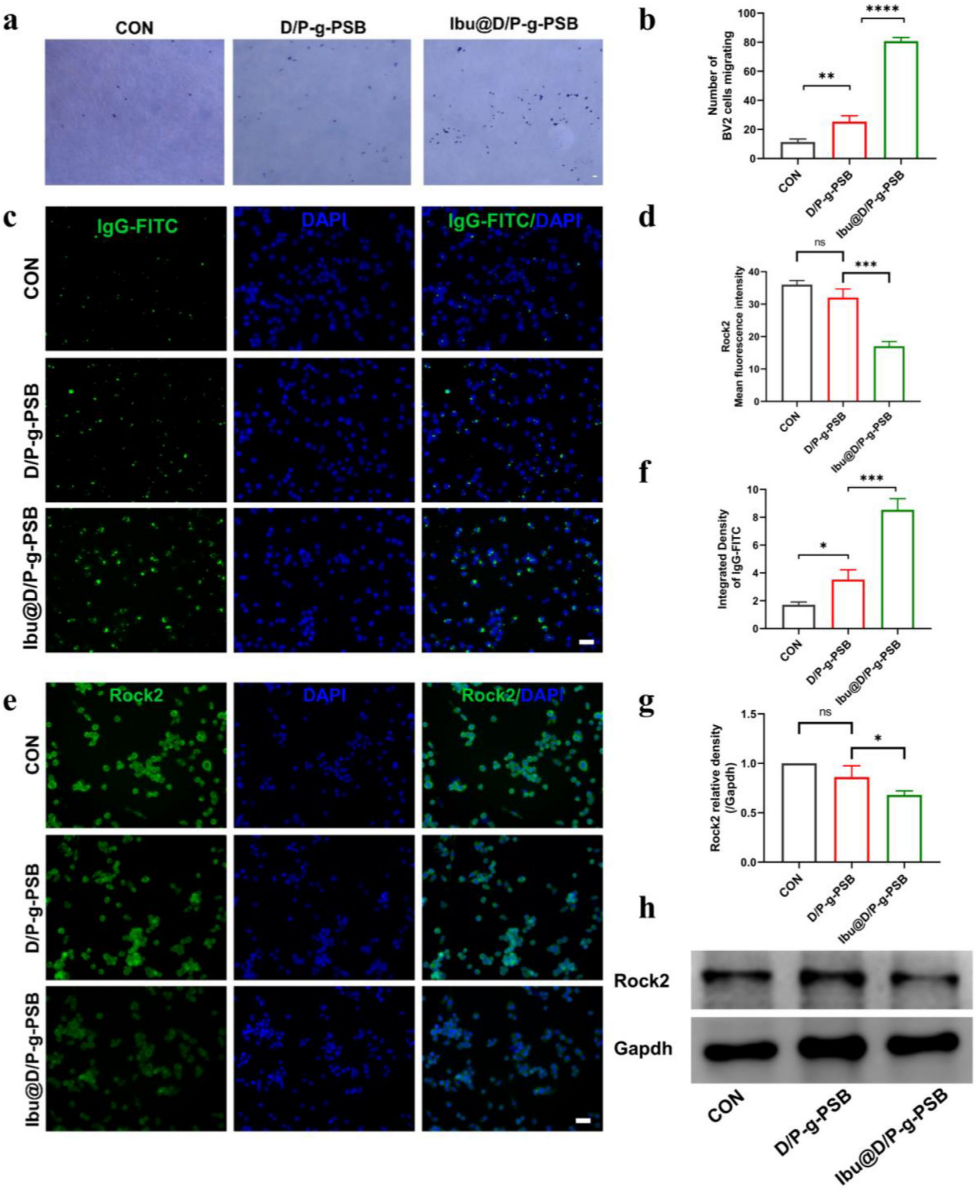
Inhibition of the RhoA/ROCK2 pathway by Ibu@D/P‐g‐PSB treatment. a) Assessment of the migratory ability of BV2 cells after Ibu@D/P‐g‐PSB treatment and its b) quantitative results (*n* = 3); c) Assessment of phagocytosis of BV2 cells after Ibu@D/P‐g‐PSB treatment and its d) fluorescence quantification results (*n* = 3),Scale bar: 50 µm; e,f) Expression of ROCK2 by different treatment groups and quantitative results (*n* = 3), Scale bar: 50 µm; g,h) Representative images of Western blot of ROCK2 by different treatment groups and quantitative results (*n* = 3). ^*^ *p* < 0.05, ^**^ *p* < 0.01, ^***^ *p* < 0.001, ^****^ *p* < 0.0001.

To validate the role of ibuprofen in inhibiting the ROCK pathway during the subacute phase of spinal cord injury, first, Iba1 and ROCK kinase phenotype expression in microglia were characterized using fluorescent staining. The staining results (Figures S12 and S13, Supporting Information) showed that activated microglia highly expressed Iba1 and ROCK2 kinase phenotypes. Under normal circumstances, microglia are in a resting state; however, they continue to move and participate in the maintenance of normal activities of the nervous system. Therefore, microglial migration plays a crucial role in the recovery of nerve function after spinal cord injury. Transwell experiments were performed to detect microglial migration after hydrogel treatment. As shown in **Figure** **4**a,b, the majority of crystal violet‐stained BV2 cells were observed in the Ibu@D/P‐g‐PSB‐treated group compared with the control and D/P‐g‐PSB groups. The results showed that the number of cells penetrating from the upper chamber to the lower chamber gradually increased, suggesting that ibuprofen promoted the migratory function of BV2 cells. The phagocytosis of BV2 cells was evaluated by simulating phagocytosis using IgG‐FITC microspheres. As shown in Figure 4c,d, Ibu@D/P‐g‐PSB‐treated BV2 cells exhibited stronger phagocytosis than the LPS group. Ibu@D/P‐g‐PSB‐treated BV2 cells showed enhanced potential to phagocytose damaged neuronal cell debris after spinal cord injury.

When spinal cord injury occurs, a large amount of myelin‐derived inhibitory factors is released around the injury, leading to the activation of RhoA protein, which transmits inhibitory signals to ROCK kinase. Activated ROCK kinase further affects downstream pathways, which in turn inhibit nerve axon regeneration, ultimately leading to nerve regeneration failure. ROCK2 expression was detected by fluorescence staining in each group. As shown in Figure 4e,f, the weakest fluorescence intensity of ROCK2 was observed in the Ibu@D/P‐g‐PSB‐treated group compared to that in the control and D/P‐g‐PSB groups. It suggested that ibuprofen could effectively inhibit the ROCK pathway, thereby reducing RhoA signaling activation, activating the transcription factor peroxisome proliferator‐activated receptor gamma (PPARγ), and reducing the deleterious cascade response after spinal cord injury.

### 2.6 Tunel Staining of Neurons In vitro

In addition, to demonstrate its potential protective effect, the OGD/R model was used to simulate biomimetic ischemic microenvironments after spinal cord injury. The level of apoptosis and the number of surviving neuronal cells have a significant impact on the prognosis of spinal cord injury. After constructing the OGD/R model, the Ibu@D/P‐g‐PSB hydrogel was co‐incubated with neural stem cells, and the results are shown in Figure S14 (Supporting Information). The number of neurons was assessed using TUNEL staining. Compared with the OGD/R group, the number of TUNEL‐positive cells was higher in the Ibu@D/P‐g‐PSB group, indicating that the number of apoptotic cells was significantly lower in the Ibu @D/P‐g‐PSB group than in the untreated and D/P‐g‐PSB hydrogel groups. These results show that ibuprofen released from the hydrogel could reduce neuronal apoptosis and effectively promote neuronal regeneration, thus reducing spinal cord injury and promoting functional recovery.

### 2.7 Assessment of the Repair of Spinal Cord Injury

#### 2.7.1 Motor Function

Preoperatively, all mice had a BMS score of 9 in both hindlimbs, normal joint movement in both hindlimbs, and normal diet and defecation. Postoperatively, all mice had a BMS score of 0 for both hindlimbs. All the mice were paralyzed in their hindlimbs and had a complete loss of voluntary defecation and urination.

**Figure 5:**
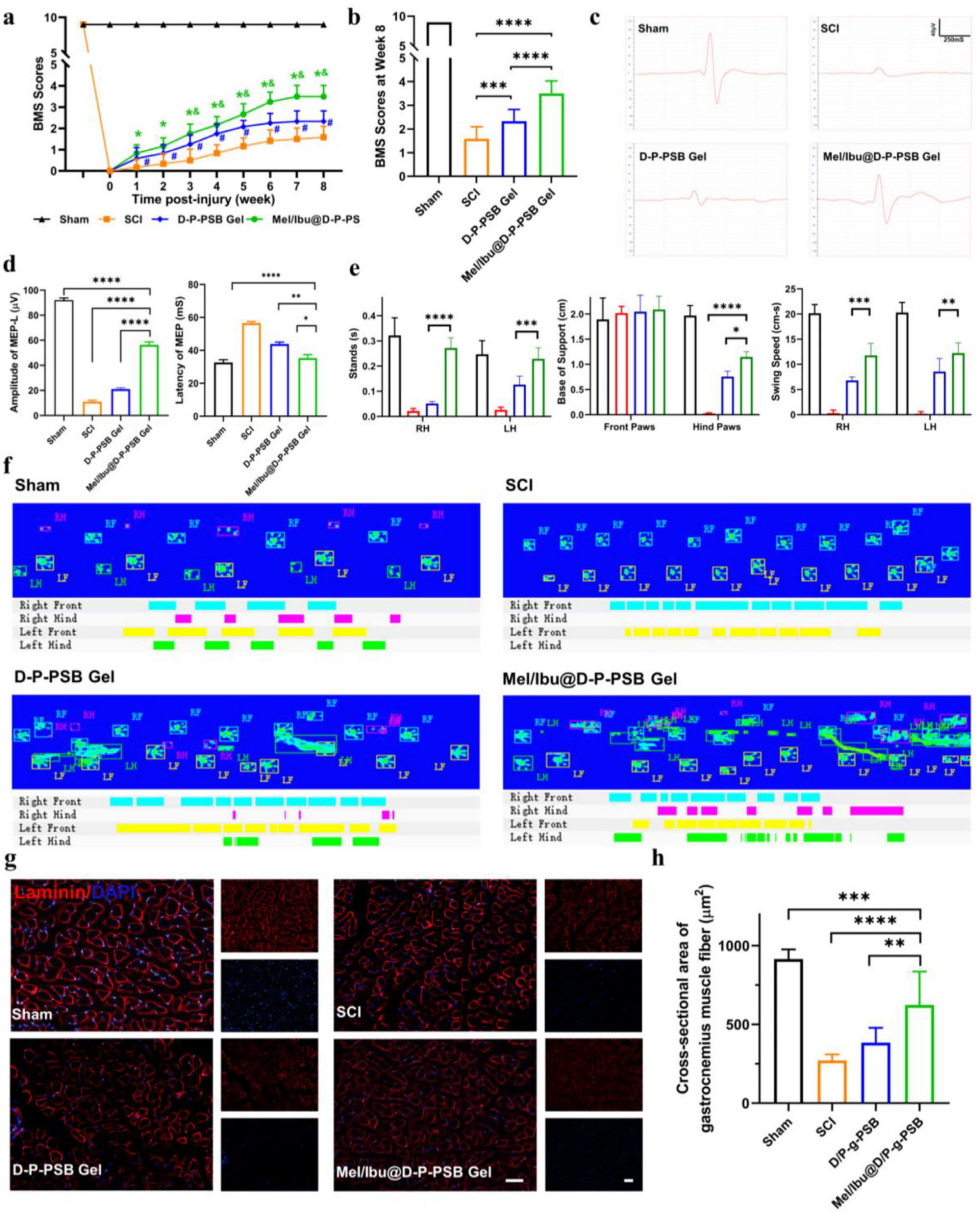
Assessment of the repair of spinal cord injury after Mel/Ibu@D‐P‐PSB hydrogel treatment. a) BMS scores on hindlimb function in mice (*n* = 6); b) BMS scores at week 8 (*n* = 6); c) Representative MEPs results of each group (*n* = 6); d) Quantitative analysis of amplitude (left) and latency (right) of MEP (*n* = 6); e,f) Gait analysis of mice (*n* = 3); g,h) Representative images of laminin staining on the gastrocnemius muscles to mark the area of muscle fibers and quantitative analysis of the cross‐sectional area of gastrocnemius muscle fibers (*n* = 10). Scale bar: 100 µm.^*^ *p* < 0.05, ^**^ *p* < 0.01, ^***^ *p* < 0.001, ^****^ *p* < 0.0001.

We used 50% Mel/Ibu@D/P‐g‐PSB hydrogel to verify its effect on spinal cord injury repair. The mice were evaluated for motor function in the 8th week after surgery, and the results (**Figure** **5**a) showed that the scores of the mice gradually increased with time. In the 8th week, as shown in Figure 5b, the mice in the Mel/Ibu@D/P‐g‐PSB hydrogel group had the best hindlimb locomotion with the highest score of 3.6±1.5, and the scores for the D/P‐g‐PSB hydrogel group were 2.5±0.8, and for the spinal cord injury group were 1.7±1.0. The D/P‐g‐PSB and Mel/Ibu@D/P‐g‐PSB hydrogel‐treated groups had significantly higher BMS scores than the SCI group. The mice in the SCI group developed severe motor dysfunction with posterior paralysis, urinary retention, and reduced feeding, requiring manual assistance for urination. The recovery of the motor function in the Mel/Ibu@D/P‐g‐PSB gel group was faster and more effective than that in the spinal cord injury group.

#### 2.7.2 Motor Evoked Potential (MEPs)

MEPs were recorded 8 weeks after injury (Figure 5c). The MEP signals in the hindlimbs in the SCI group were significantly weaker than those in the hydrogel group. The Mel/Ibu@D/P‐g‐PSB hydrogel group exhibited waveforms similar to those of the Sham group, suggesting that the motor functions of the hindlimb were partially restored, although the amplitude was lower than that of normal C57 mice. Quantification of the amplitude and latency in the different groups is shown in Figure 5d, where Mel/Ibu@D/P‐g‐PSB hydrogel‐treated mice exhibited a greater amplitude and shorter latency than the SCI mice. These results indicate that the Mel/Ibu@D/P‐g‐PSB hydrogel effectively restores motor function in mice and promotes functional recovery after spinal cord injury.

#### 2.7.3 Gait Analysis

The performance of the hindlimbs during locomotion was analyzed to assess the recovery of hindlimb locomotor ability, as shown in Figure 5e,f, and Figure S15 (Supporting Information). Compared to the mice in the Sham group, which walked with their hindlegs propped up in the field, the mice in the SCI group walked on their front legs more pragmatically, indicating that the mice were deficient in hindlimb locomotor ability. The results showed that mice recovered their hindlimb locomotor ability and were able to use their hind limbs to walk after Mel/Ibu@D/P‐g‐PSB hydrogel treatment.

### 2.8 Histological Analysis

After spinal cord injury, muscles often experience disuse atrophy and decreased muscle circumference owing to the absence of CNS control. To investigate whether the Mel/Ibu@D/P‐g‐PSB hydrogel can reduce muscle atrophy after spinal cord injury in mice, the area of muscle fibers was measured, and the degree of atrophy was assessed by staining the gastrocnemius muscle with laminin. Compared with the Sham group, the cross‐sectional area of gastrocnemius muscle fibers in the hindlimbs of mice in the SCI group was significantly reduced due to atrophy (Figure 5g). The muscle fibers of mice in the Mel/Ibu@D/P‐g‐PSB hydrogel group were thicker, and their cross‐sectional area was increased compared with that of the SCI group (Figure 5h), which further demonstrated that Mel/Ibu@D/P‐g‐PSB hydrogel treatment could improve muscle locomotor function in the hindlimb with spinal cord injury.

Spinal cord injuries, particularly irreversible injuries arising from the lower lumbar segment of the spinal cord, can lead to neurogenic bladder (NB). Some studies have shown that the incidence of NB in patients with spinal cord injury is ≈70%–84%, and most cases of NB caused by spinal cord injury manifest as urinary retention and dysfunction of bladder contraction of the forced urethral muscle. Therefore, bladder function is an important factor in the evaluation of spinal cord injury repair. Spinal cord injury can cause a certain degree of cystic fibrosis in the absence of effective treatment after NB. We assessed the effectiveness of each group in the treatment of spinal cord injury by H&E staining of the bladder. As shown in Figure S16 (Supporting Information), in both experiments, to validate anti‐inflammation and verify spinal cord repair, compared to the control group, after spinal cord injury, the normal fibrous connective tissue in the bladder wall of the mice in the SCI group was gradually disordered. Over time, the thickness of the bladder wall changed, the lamina propria decreased, the smooth muscle hypertrophied and thickened, the intermuscular collagen fibers gradually increased, and the staining became darker. Collagen fibers in the bladders of mice after Mel@D/P‐g‐PSB and Mel/Ibu@D/P‐g‐PSB hydrogel treatment were significantly lower than those in the SCI group. After drug treatment, spinal cord injury was repaired to some extent, and bladder function was restored to some extent in all mice.

### 2.9 Mel/Ibu@D/P‐g‐PSB Hydrogel Reduces Inflammatory Response during the Inflammatory Response Phase In vivo

**Figure 6:**
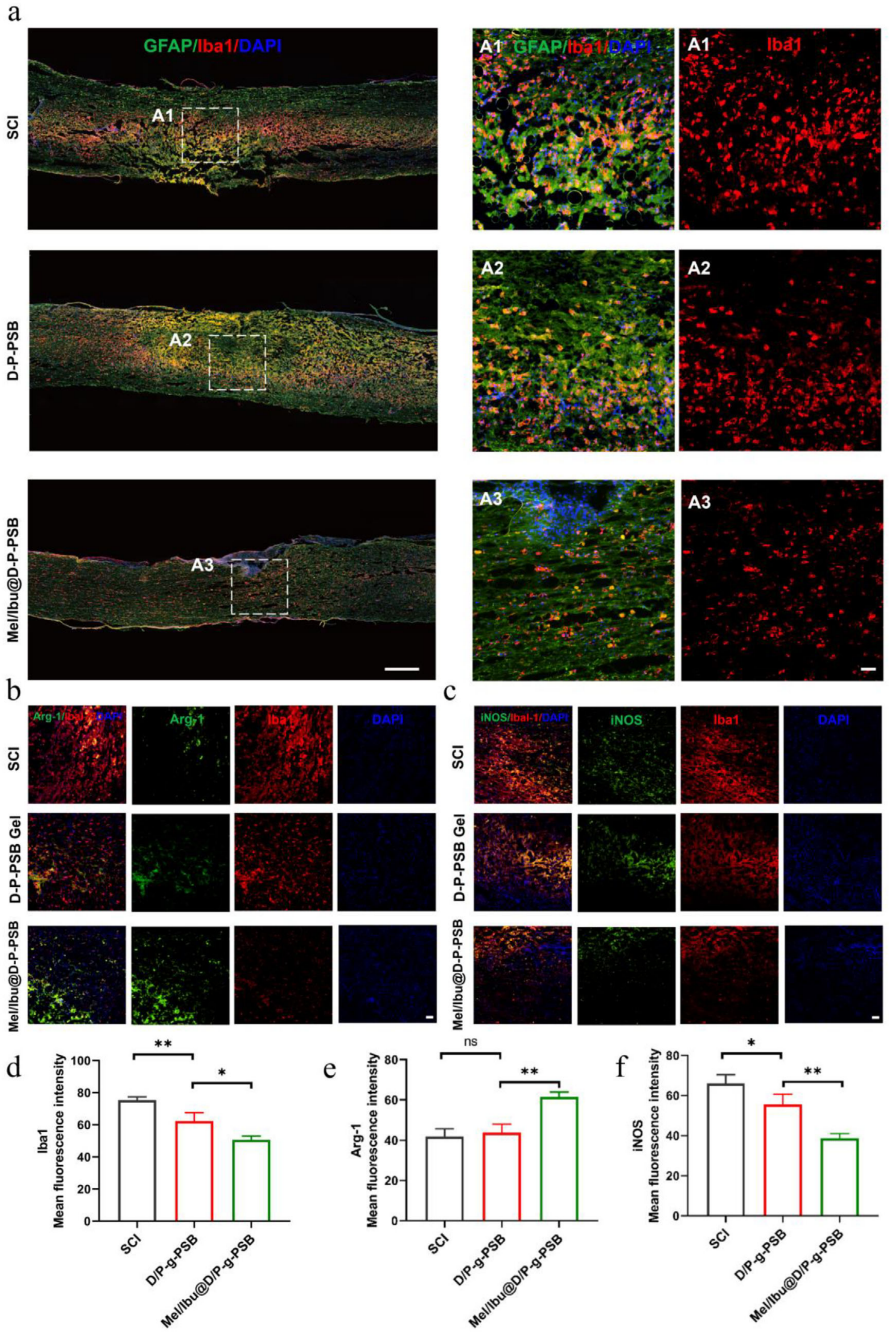
Mel/Ibu@D/P‐g‐PSB hydrogel reduces inflammatory response during the acute phase in vivo. a) Representative immunofluorescence images of Iba1 and GFAP at the injury site at the eighth week after spinal cord injury(left image: scale bar 500 µm; right image: scale bar 50 µm). Immunofluorescence images of Iba1/ Arg‐1b) and Iba1/iNOS c) at week 8 after spinal cord injury (scale bar 50 µm). Fluorescence quantification results for different cells: d) Iba1‐positive cells, e) Arg‐1‐positive cells, and f) iNOS‐positive cells. (*n* = 3). ^*^ *p* < 0.05, ^**^ *p* < 0.01, ^***^ *p* < 0.001, ^****^ *p* < 0.0001.

Inhibiting the inflammatory response and promoting microglial M2 polarization in the early stages of spinal cord injury can attenuate damage after spinal cord injury, reduce neuronal apoptosis, and create a favorable microenvironment for the repair of spinal cord injury. Therefore, we used immunofluorescence staining to detect the fluorescence intensities of Iba1, Arg‐1, and i‐NOS, which are indicators of inflammation at the site of spinal cord injury. Immunofluorescence staining of Iba1 and GFAP was performed to assess the inflammation‐reducing effects. A significant reduction of Iba1‐positive cells was observed in the hydrogel group (**Figure** **6**a,d). The Mel/Ibu@D/P‐g‐PSB hydrogel extended‐release of melatonin and ibuprofen reduced inflammation during the acute phase of spinal cord injury in mice. The fluorescence staining results for Iba1/iNOS and Iba1/Arg‐1 showed that, in the region of spinal cord injury after spinal cord injury, the fluorescence intensity of iNOS was stronger than that of the other treatment groups (Figure 6b,e), and the fluorescence intensity of Arg‐1 was weaker than that of the other treatment groups (Figure 6c,f). This suggested that Mel/Ibu@D/P‐g‐PSB hydrogel therapy could improve the immune microenvironment by promoting microglial polarization toward the M2 type and inhibiting M1 polarization, thus repairing and remodeling the injured tissue. The level of apoptosis or the number of surviving neuronal cells in a large number of neurons has an important impact on the prognosis of spinal cord injury. As shown in Figure S17 (Supporting Information), the number of TUNEL‐positive cells in the model group was significantly higher than that in the blank gel and Mel/Ibu@D/P‐g‐PSB hydrogel groups. These results indicated that the hydrogel could provide adhesion sites for neuronal growth, promote neuronal proliferation and differentiation, and reduce apoptosis. On the other hand, the drug treatment improved the pathological microenvironment of spinal cord injury tissues. It could regulate the pro‐inflammatory microenvironment, inhibit the activation of factors related to the expression of the NF‐κB signaling pathway, and reduce the production of pro‐inflammatory cytokines, thus reducing inflammation, exerting neuroprotective effects, and ultimately effectively protecting nerve cells.

### 2.10 Mel/Ibu@D/P‐g‐PSB Hydrogel Promotes Recovery from Spinal Cord Injury In vivo

**Figure 7:**
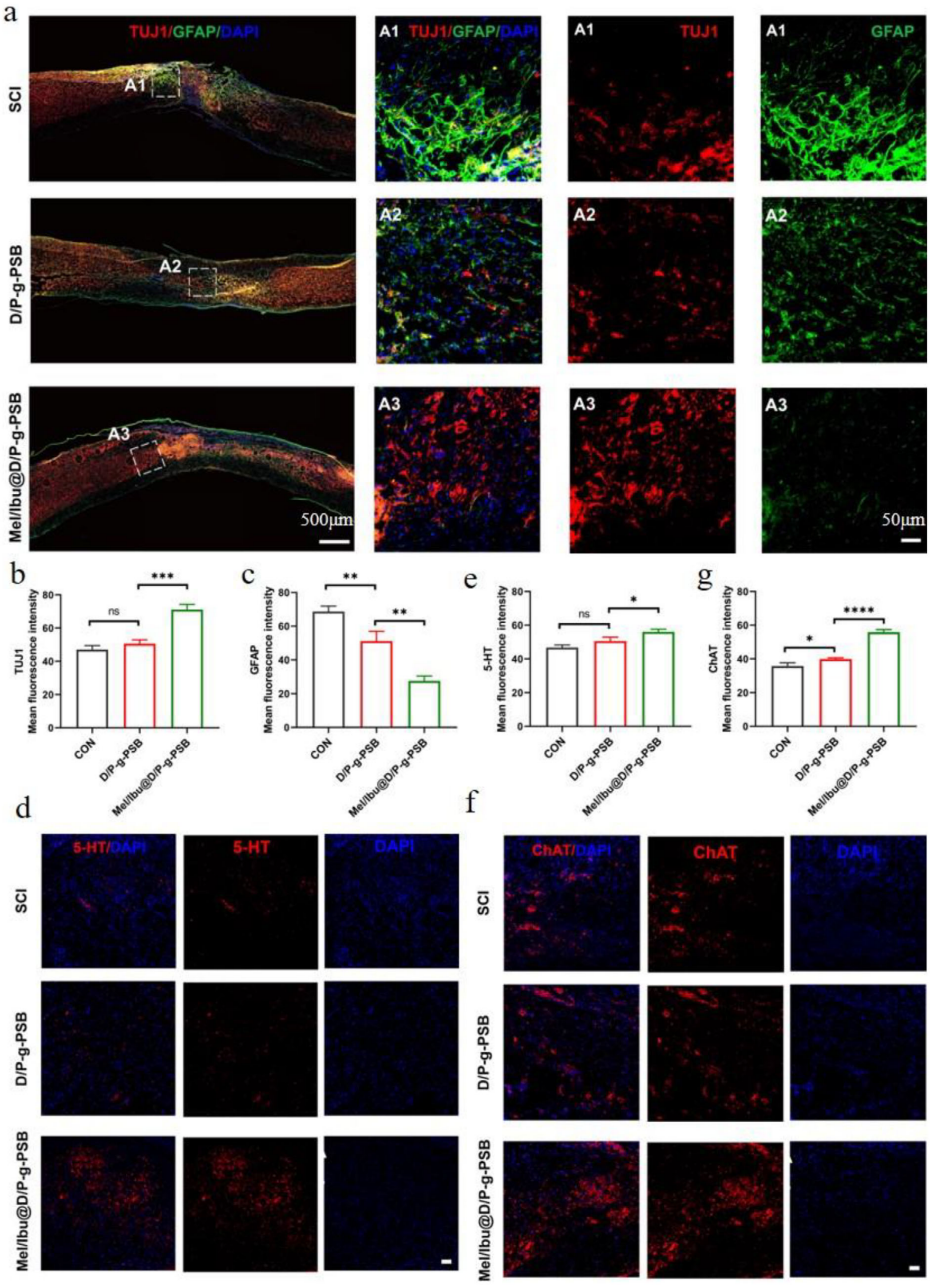
Mel/Ibu@D/P‐g‐PSB hydrogel promotes neuronal differentiation and inhibits astrocytes. a) Representative immunofluorescence images of Tuj 1 and GFAP at the injury site.(left image: scale bar 500 µm; right image: scale bar 50 µm;) Fluorescence quantification assay of b) Tuj 1 and c) GFAP, respectively. d) Representative immunofluorescence images of d) 5HT and f) ChAT at the injured area. Scale bar: 50 µm. Fluorescence quantification assay of e) 5HT and (f) ChAT. (*n* = 3). ^*^ *p* < 0.05, ^**^ *p* < 0.01, ^***^ *p* < 0.001, ^****^ *p* < 0.0001.

In the CNS, astrocytes favor the elimination of inflammation and promote the repair of spinal cord injury. However, excessive astrocytic proliferation is a typical response to trauma and inflammation. Increased GFAP expression is one of its main features. Tuj‐1 is also a marker of early neurons, and its expression responds to neuronal survival and spinal cord recovery.^[^ ^37^ ^]^ To assess the neuronal differentiation after Mel/Ibu@D/P‐g‐PSB hydrogel treatment, we double‐stained the spinal cord tissue with GFAP (green) and Tuj‐1 (red) (**Figure** **7**a,b). The proportion of Tuj‐1‐positive cells differed significantly among groups. The proportion of Tuj‐1‐positive cells was significantly higher in the D/P‐g‐PSB gel and Mel/Ibu@D/P‐g‐PSB hydrogel groups than in the SCI group, whereas the proportion of Tuj‐1‐positive cells in the Mel/Ibu@D/P‐g‐PSB hydrogel group was significantly higher than that in the D/P‐g‐PSB hydrogel group. This indicated that the Mel/Ibu@D/P‐g‐PSB hydrogel promoted neuronal survival in the injured region. A hydrogel with a 3D mesh structure mimics the extracellular matrix, providing adhesion sites for neuronal cells and promoting the proliferation and differentiation of neurons in the injured region. Compared with the SCI group, the number of GFAP‐positive cells in the Mel/Ibu@D/P‐g‐PSB hydrogel group was significantly reduced, and the quantitative results (Figure 7b) showed that the intensity of red fluorescence in the SCI group was higher than that in the Mel/Ibu@D/P‐g‐PSB hydrogel group. It was also suggested that Mel/Ibu@D/P‐g‐PSB hydrogel treatment significantly inhibited astrocyte overexpression, reduced the severity of initial reactive gliosis, and suppressed glial scarring.

5‐HT is a neuromodulator of the CNS, and the central gray matter of the spinal cord contains abundant 5‐HTergic nerve endings. Fluorescence staining (Figure 7c,d) showed that the density of 5‐HT fibers at the lower end of the spinal cord after spinal cord injury was significantly lower than that in the normal and hydrogel groups. Increased 5‐HT levels can help promote the recovery of motor function in mice with spinal cord injury.

To evaluate the recovery of the motor axons, localization analyses were performed (Figure 7e,f). The fluorescence staining results showed that at 8 weeks after transplantation, the fluorescence intensity of ChAT at the injury site was significantly higher in both hydrogel groups than in the SCI and D/P‐g‐PSB hydrogel groups. These results indicate that after hydrogel implantation, a neural network was established at the injury site, providing a structural basis for physiological function.

These results showed that the Mel/Ibu@D/P‐g‐PSB hydrogel promoted spinal cord repair after spinal cord injury.

### 2.11 The Mechanism of Mel/Ibu@D/P‐g‐PSB Hydrogel Improving the Microenvironment In vivo

**Figure 8:**
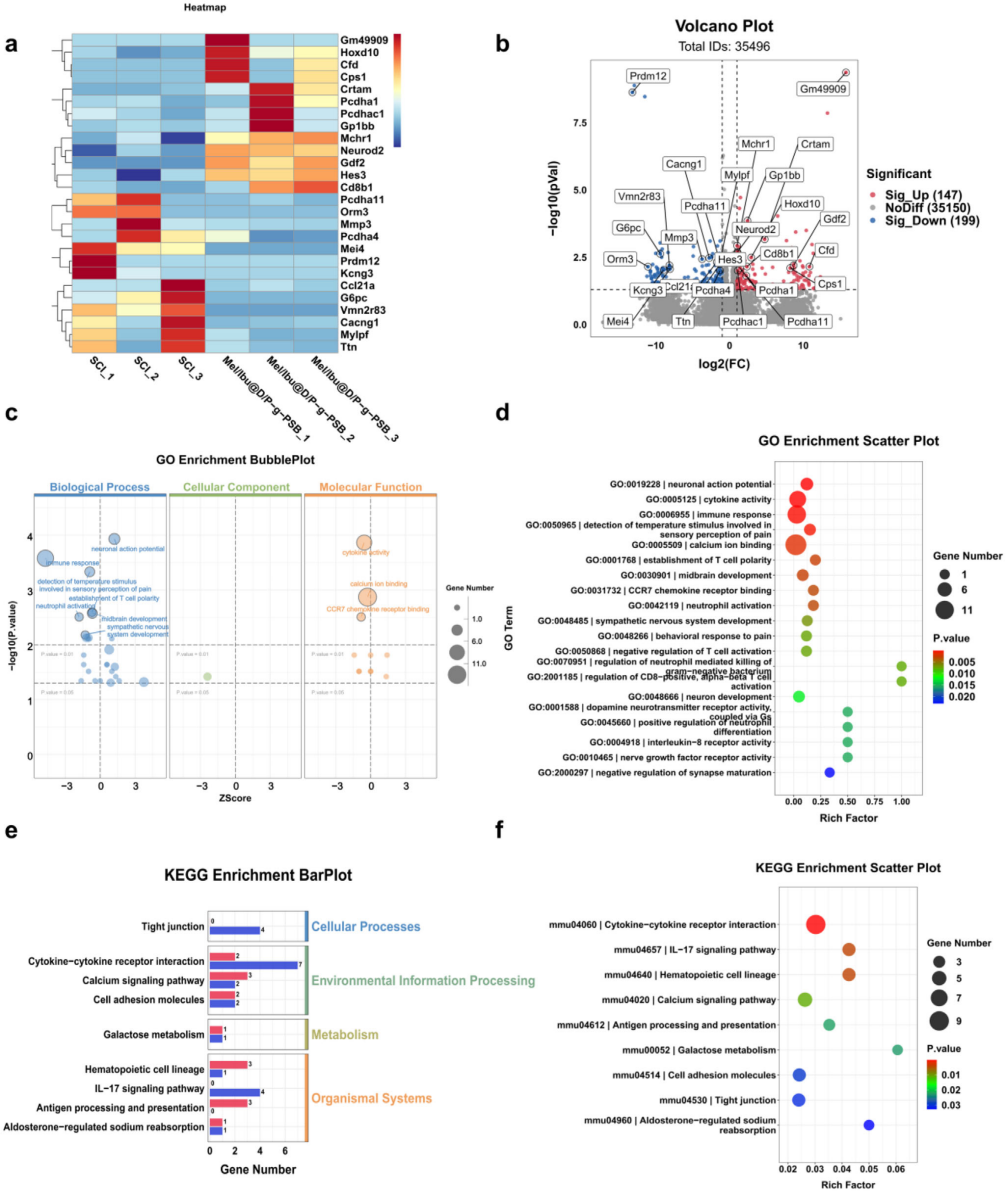
The mechanism of Mel/Ibu@D/P‐g‐PSB hydrogel improving the microenvironment in vivo. a) The heat map of genes of interest with significant differences. b) The gene volcano map shows the expression of these genes. c) The bubble plot diagram of GO enrichment. d) The scatter plot diagram of GO enrichment. e) The bar plot of KEGG enrichment. f) The scatter plot of KEGG enrichment shows the KEGG pathways.

RNA‐sequencing analysis was performed to investigate the mechanism of action of the Mel/Ibu@D/P‐g‐PSB hydrogel in modulating the microenvironment during the subacute phase of SCI. In the heat map (**Figure** **8**a), we visualized the expression profiles of differentially expressed genes (DEGs) related to immune response, regulation of immune system processes, and regulation of neuron differentiation between the Mel/Ibu@D/P‐g‐PSB treatment group and the SCI group. The color gradient (red indicating upregulated genes and blue indicating downregulated genes) represented the log2 fold change of gene expression, which revealed distinct expression patterns of genes involved in these functional categories. Genes with significant differences were screened in the heat map related to the immune response, regulation of immune system processes, and regulation of neuron differentiation (Figure 8a). A gene volcano map showed the expression of the genes of interest (Figure 8b). For functional enrichment analysis, the bubble plot of Gene Ontology (GO) enrichment (Figure 8c) categorized DEGs into three ontologies: Biological Process (BP), Cellular Component (CC), and Molecular Function (MF). Bubbles varied in size according to the number of enriched genes, and their positions reflected the enrichment significance. In the BP ontology, DEGs were enriched in immune‐related processes (like immune response and regulation of immune system processes) and neuron development‐related processes (such as neuron development and axonogenesis). The CC ontology highlighted enrichment in cellular components associated with immune or neural structures, while the MF ontology indicated enrichment in functions such as cytokine activity and chemokine receptor binding. The scatter plot of GO enrichment (Figure 8d) displayed individual GO terms. Key GO terms relevant to microenvironment improvement were identified: cytokine activity (critical for immune cell communication and inflammation modulation), immune response (regulating leukocyte infiltration and cytokine secretion), neutrophil activation (a key event in subacute inflammation), CCR7 chemokine receptor binding (governing immune cell migration), regulation of CD8 – positive cells (balancing cytotoxic and regulatory immune responses), alpha – beta T – cell activation (adaptive immune regulation), neuron development (supporting neural cell survival and differentiation), and axonogenesis involved in innervation (facilitating neural circuit reconstruction). These enrichments suggest that the hydrogel modulates transcriptional programs underlying both immune homeostasis and neural regeneration, two pillars of spinal cord microenvironment remodeling. The bar plot of KEGG enrichment (Figure 8e) grouped DEGs into functional categories, including Environmental Information Processing, Organismal Systems, Metabolism, and Cellular Processes. The length of each bar indicated the number of enriched KEGG pathways within each category, demonstrating that the hydrogel‐induced transcriptional changes span multiple biological dimensions—from immune signaling and neural transmission to metabolic and cellular homeostasis. The scatter plot of KEGG enrichment (Figure 8f) visualized individual KEGG pathways. Pathways integral to microenvironment improvement included cytokine–cytokine receptor interaction (the core network for immune cell crosstalk and cytokine balance), IL‐17 signaling pathway (regulating pro‐inflammatory cascades and tissue repair dynamics during subacute SCI), and calcium signaling pathway (orchestrating neuronal excitability, gene transcription, and cell survival—key for neural repair microenvironment). These pathway enrichments imply that the hydrogel reshapes the microenvironment by regulating immune cell‐derived cytokine networks, dampening excessive inflammation, and activating neural regenerative signals. Collectively, these RNA–seq data demonstrate that Mel/Ibu@D/P‐g‐PSB modulates the subacute SCI microenvironment through coordinated transcriptional regulation of immune–inflammatory responses and neural regenerative programs.

## 3 Discussion

Spinal cord injury is a severe disease caused by multiple and complex pathological processes, including neuronal apoptosis, glial cell proliferation, glial scarring, and impeded axonal regeneration.^[^ ^1^, ^2^ ^]^ In this study, we designed a Mel/Ibu@D/P‐g‐PSB hydrogel with sequential drug release to maximize therapeutic efficacy during the recovery period of spinal cord injury. Melatonin and ibuprofen are loaded in the D/P‐g‐PSB hydrogel by different mechanisms: the physically blended melatonin achieves early and rapid release through diffusion effect, while the ibuprofen with charges linked to PSB segments in the hydrogel backbone through electrostatic interaction can prolong the released period as the hydrogel degradation. Here, the Per‐g‐PSB nanogel, a four‐armed dendritic macromolecule, was not only used to adjust the mechanical properties of the Dex‐g‐PSB hydrogel, but also provided the drug loading sites. As a result, drug‐polymer network interactions were established in this system, which is critical for sequential drug release.^[^ ^38^ ^]^ Moreover, traditional nanoparticle hybrid hydrogels are heterogeneous hydrogels that often have a heterogeneous polymer network, which affects their mechanical strength and deformation properties.^[^ ^20^ ^]^ Our hydrogel system has the same polymer pSBMA segments, which have better mechanical properties and a homogeneous internal structure, resulting in higher biocompatibility. This multifunctional therapy is important for clinical treatment and provides new therapeutic insights into spinal cord injury repair.

In the acute phase, melatonin promotes the conversion of microglia from the M1‐type to the M2‐type by decreasing the expression and release of pro‐inflammatory mediators, thereby reducing spinal cord injury and promoting the recovery of motor function. In the chronic phase, ibuprofen acts as a ROCK pathway inhibitor to reduce RhoA signaling activation and activate the transcription factor peroxisome PPARγ, which reduces the deleterious cascade response after spinal cord injury. Therefore, this nanogel‐incorporated injectable hydrogel system showed significantly improved effects on the complete transection injury of the spinal cord in vivo.

## 4 Conclusion

In this study, we synthesized an ion‐sensitive injectable physical hydrogel based on dextran and pentaerythritol. Per‐g‐PSB, a dendritic macromolecule, was introduced into Dex‐g‐PSB as a cross‐linker to adjust the mechanical properties of Dex‐g‐PSB to match the tissue. Two drugs, melatonin and ibuprofen, were loaded onto the gel to form a Mel/Ibu@D/P‐g‐PSB hydrogel via two different mechanisms. Because the synthesized D/P‐g‐PSB polymer was self‐assembled to form a hydrogel based on electrostatic forces, uncharged melatonin was physically mixed in the hydrogel, which could be released first by diffusion. It exerts anti‐inflammatory and antioxidant effects during the pre‐injury stage of spinal cord injury, thus reversing macrophages from the M1 to the M2 phenotype and promoting functional recovery. Ibuprofen is inherently negatively charged and interacts electrostatically with the positively charged Per‐g‐PSB nanogel skeleton, showing significant sustained‐release properties compared with melatonin. In the later stages of recovery, it blocks the ROCK pathway and inhibits RhoA signaling activation, leading to activation of the transcription factor PPARγ and reducing the deleterious cascade of responses. Based on the results of the above in vivo and in vitro tests, Dex/Per‐g‐PSB hydrogel with good biocompatibility demonstrated an excellent repairing effect on spinal cord injury and promoted nerve regeneration.

## 5 Experimental Section

### Materials

Pentaerythritol (Per, 99%), 2‐bromoisobutyryl bromide (BIBB, 98%), 2‐(dimethylamino) ethyl methacrylate (DMAEMA, 99%), Dextran (Dex, MW40000), 1,3‐propanesulfonate (99%), 2,2‐Bipyridyl (99%), and copper(I) bromide (CuBr, 99%) were purchased from Aladdin. Triethyiamine (99%), N, N‐dimethylformamide (99.5%), and Dimethyl sulfoxide (DMSO, 99.5%) were purchased from Tianjin Fuyu Fine Chemical Company. BCA kit, RIPA lysis buffer, protease inhibitor and phosphatase inhibitor were purchased from Shanghai Beyotime Biotechnology Co. Cell counting kit‐8 (CCK8), Calcein AM/PI Live/Dead cell double staining kit and RNA extraction kit were purchased from Beijing Solarbio Science & Technology Co., Ltd. RMPI 1640 and DMEM‐HG were purchased from Thermo Fisher Scientific Inc. PrimeScriptTM RT reagent kit was purchased from Takara Biomedical Technology (Beijing) Co., Ltd. Anti‐ iNOS (22226‐1‐AP, Proteintech, the US), anti‐Arg‐1 (66129‐1‐Ig, Proteintech, the US), and β‐actin (HRP‐60008) were purchased from Proteintech Group. Inc (The US).

### Synthesis of the Per‐Br

Pentaerythritol was dissolved in 20–30 mL of THF and stirred for some time, after which triethylamine and 2‐bromoisobutyl bromide (14.72 g, 64 mmol) were added. Thereafter, the reaction mixture was placed in an ice bath for 2 h and then under a nitrogen atmosphere at room temperature for 24 h. After the completion of the reaction, ether was added for extraction. Subsequently, the mixture was washed with 10% hydrochloric acid solution, saturated sodium carbonate solution, and water. The obtained organic phase was dried with anhydrous magnesium sulfate and filtered. The crude product was obtained by spin evaporation, and the final white solid product was obtained by recrystallization with methanol.

### Synthesis of Per‐g‐PSB

Per‐Br (0.05 g) was weighed and dissolved in DMSO, and a mixed solution of SBMA/H_2_O (100 mmoL) was added. To catalyze the reaction, 3 mmol of copper bromide (CuBr) and 4.5 mmol of 2,2'‐bipyridyl (bpy) were introduced. Then the reaction was carried out under nitrogen protection for 24 h. Subsequently, the product was precipitated in methanol and purified.

### Synthesis of Dex/Per‐g‐PSB

Dex‐g‐PSB was synthesized according to the method described in the previous study. The Dex‐g‐PSB and Per‐g‐PSB polymer powders were briefly mixed. Saline was added to obtain a physical hydrogel based on the formulations listed in Table S2 (Supporting Information).

### Characterization of Dex/Per‐g‐PSB—^1^H‐NMR and FT‐IR

Per‐Br and Per‐g‐PSB were dissolved in DMSO‐d6 and characterized using proton nuclear magnetic resonance (^1^H‐NMR) spectroscopy (Bruker DMX‐400 spectrometer). Per‐Br and Per‐g‐PSB were characterized using Fourier transform infrared (FT‐IR) spectroscopy (Nicolet iN10 Infrared Microscope, U.S.A.).

### Characterization of Dex/Per‐g‐PSB—SEM

The inner morphologies of the dried hydrogels were studied using scanning electron microscopy (SEM, S‐2500, Hitachi Seiki, Tokyo, Japan).

### Characterization of Dex/Per‐g‐PSB—Rheological Assay

The rheological characterization of D/P‐g‐PSB hydrogels was performed using a control‐strain rheometer (TA Instruments Inc., New Castle, DL) under 1% strain stress and 0.1–30 Hz frequency.

### Characterization of Dex/Per‐g‐PSB—Swelling Ratio

The dry Dex/Per‐g‐PSB hydrogels were fully immersed in deionized (DI) water and saline to reach swelling equilibrium. The swelling ratios of the hydrogels were calculated using the following formula:

(1) $$\mathrm{Swelling}\;\mathrm{ratio}\;\left(\%\right)=\frac{W0-W1}{W1}\times 100\%$$

### Characterization of Dex/Per‐g‐PSB—In vitro Degradation

The in vitro degradation of Dex/Per‐g‐PSB hydrogels was investigated by measuring their weight loss over time. Briefly, 2 g of the hydrogel (W0) was immersed in 20 mL of DI water or phosphate‐buffered saline (PBS; 10 mm, pH 7.4). At predetermined time points, the hydrogel was removed, weighed (W1), and calculated as follows:

(2) $$\mathrm{Degradation}\;\mathrm{rate}\;\left(\%\right)=\frac{W0-W1}{W1}\times 100\%$$

### Characterization of Dex/Per‐g‐PSB—In vitro Drug Release

PBS solution was used to assess the release behavior of melatonin and ibuprofen from the hydrogels. Hydrogels were prepared directly in 15 mL centrifuge tubes, to which 5 mL of PBS was added. The system was placed in a thermostatic water bath shaker (temperature maintained at 37 °C) and incubated with oscillations at a speed of 100 rpm. At the predetermined time points, 1 mL of the medium was collected, and the released melatonin and ibuprofen were determined by high‐performance liquid chromatography (HPLC).

### Blood Compatibility

The hydrogel was placed in saline for 4 h, and the supernatant was collected to obtain the hydrogel extract. Blood was collected from the carotid sinus of mice and centrifuged to obtain the accumulated red blood cells (RBCs), which were added to saline to prepare a 2% RBC suspension. The RBCs were incubated with different hydrogel extracts for 4 h at 37 °C. Saline and DI water were used as negative and positive controls, respectively. The absorbance of the supernatant (Asample) was measured at 541 nm, and the hemolysis rate was calculated using the following formula:

(3) $$\mathrm{Hemolysis}\;\;\left(\%\right)=\frac{\mathrm{Asample}-\mathrm{Anegative}\;\mathrm{control}}{\mathrm{Apositive}\;\mathrm{control}-\mathrm{Anegative}\;\mathrm{control}}\times 100\%$$

### Protein Resistance Evaluation

Bovine serum albumin (BSA) was chosen as the model protein to study the anti‐protein adsorption properties of the hydrogels. The hydrogels were immersed in 1.5 mL of BSA solution (2 mg mL^−1^) after reaching swelling equilibrium and incubated at 37 °C for 4 h. The hydrogels were washed three times with saline to remove the unadsorbed proteins. Finally, the protein content of the eluate was determined using a bicinchoninic acid (BCA) kit.

### In vitro Cell Experiments—Cytotoxicity Test of Hydrogels

The cytotoxic effects of the hydrogel on L929 cells and BV2 cells were evaluated using the CCK‐8 assay. The hydrogel extract was prepared by soaking 0.5 mL of hydrogel in the RPMI‐1640 medium(Gibco, the US). L929 cells and BV2 cells were seeded in 96‐well plates at a density of 1 × 10^4^ and incubated at 37 °C under 5% CO_2_. After 12 h, the upper medium was replaced with 100 µL of hydrogel extract and incubated for 24 and 48 h, respectively. Thereafter, the cells were washed with PBS (Gibco, the US) three times and then replaced with 100 µL of new culture medium containing 10% v/v CCK‐8. The cells were incubated at 37 °C for 2 h under dark conditions; their absorbance at 450 nm was measured with a microplate reader, and the cell survival rate was calculated.

### In vitro Cell Experiments—3D Culture

BV2 cells were resuspended in a culture medium to obtain a cell suspension at a density of 8 × 10^6^ cells mL^−1^. The cell suspension was mixed with hydrogel powder in a 24‐well plate to obtain a hydrogel encapsulating the BV2 cells. The cells were cultured in the hydrogel for 1, 2, and 3 days, and the medium was changed daily. Buffer containing live/dead reagent was added and then co‐incubated with the hydrogel at 37 °C. After 20 min of incubation, excess dye was removed from the upper layer, and fluorescence images were captured using a confocal microscope (OLYMPUS, IX73‐DP80, Japan). The fluorescence images were quantified using Image J software to determine the changes in cell number and viability.

### In vitro Cell Experiments—Anti‐Inflammatory Analysis

Transwell chambers were used to simulate the inflammatory environment in vitro to investigate the protective effect of Mel@D/P‐g‐PSB hydrogels against oxidative damage during the inflammatory process of spinal cord injury. BV2 cells were grown in six‐well plates at a density of 2 × 10^4^ cells well^−1^. Inflammation was induced by replacing the normal medium with a serum‐free medium containing 200 ng mL^−1^ lipopolysaccharide (LPS). Mel@D/P‐g‐PSB hydrogel (300 µL) was placed in the upper chamber of the Transwell, and 1 mL of serum‐free DMEM medium was added. After one day of culture, the lower layer of the cells was collected. The expression of pro‐inflammatory M1‐related genes (iNOS, TNF‐α, and IL‐6) and anti‐inflammatory M2‐related genes (Arg‐1, CD206, and IL‐4) was detected by PCR. The fluorescence intensities of the pro‐inflammatory marker iNOS and the anti‐inflammatory marker Arg‐1 were detected by immunostaining. The fluorescence intensities of iNOS and Arg‐1 were quantified using Image J software. The protein expression of the pro‐inflammatory marker iNOS and the protein expression of the anti‐inflammatory marker Arg‐1, as well as the protein expression of NF‐κB, were detected by western blotting to ascertain whether the anti‐inflammatory effect of the hydrogel was related to the inhibition of the NF‐κB pathway. The primary antibodies used included: iNOS (22226‐1‐AP, Proteintech, the US), Arg‐1 (66129‐1‐Ig, Proteintech, the US), iba1 (17198S, CST, the US), iba1 (ab283319, abcam, the US), β‐actin (HRP‐60008, Proteintech, the US). The secondary antibodies used included: HRP‐conjugated Affinipure Goat Anti‐Mouse IgG(H+L) (SA00001‐1, Proteintech, the US), HRP‐conjugated Affinipure Goat Anti‐Rabbit IgG(H+L) (SA00001‐2, Proteintech, the US), CoraLite488‐conjugated Goat Anti‐Mouse IgG(H+L) (SA00013‐1, Proteintech, the US), CoraLite594‐conjugated Goat Anti‐Rabbit IgG(H+L) (SA00013‐4, Proteintech, the US).

### In vitro Cell Experiments—Identification of Markers for BV2 Cells

BV2 cells were grown at a density of 1 × 10^5^ cells well^−1^ in 24‐well plates and incubated at 37 °C with 5% CO_2_ for 24 h. The medium was discarded, washed with PBS, fixed with 4% paraformaldehyde (PFA) for 10 min, incubated with 0.1% Triton X‐100 for 10 min, and incubated with 5% BSA for 30 min. Anti‐Iba1 (dilution 1:300) and anti‐Rock2 (dilution 1:300) were added, and then they were incubated with anti‐Rock2 (dilution 1:200) at 4 °C overnight. The secondary antibody, Goat Anti‐Rabbit IgG H&L (AlexaFluor594) (dilution 1:600), was added and incubated at room temperature in the dark for l h. The expressions of Iba1 and Rock2 were observed using confocal laser scanning microscopy (CLSM). The primary antibodies used included: Rock2(ab45171, abcam, the US). The secondary antibodies used included CoraLite594‐conjugated Goat Anti‐Rabbit IgG(H+L) (SA00013‐4, Proteintech, the US).

### In vitro Cell Experiments—Evaluation of the Migration Function of BV2 Cells

Migration function of the Ibu@D/P‐g‐PSB hydrogel on BV2 cells during spinal cord injury was investigated using the Transwell assay. BV2 cells were grown in the upper layer of the chamber, and the lower layer was incubated for 24 h with fresh medium and D/P‐g‐PSB and Ibu@D/P‐g‐PSB hydrogels. Cells were collected and stained with 1% crystal violet to assess their migratory ability.

### In vitro Cell Experiments—Evaluation of the Phagocytic Capacity of Microglia

BV2 cells were grown in 24‐well plates at a density of 1 × 10^5^ cells well^−1^ and incubated for 12 h to allow adherence. They were then incubated with fresh medium and D/P‐g‐PSB and Ibu@D/P‐g‐PSB hydrogels for 24 h. Then, lgG‐FlTC fluorescent microspheres were added and co‐incubated at 37 °C for 3 h. Cell nuclei were stained with DAPI. The number of phagocytosed cells was observed using a confocal Ingenium light microscope and quantitatively analyzed using Image J.

### In vitro Cell Experiments—Evaluation of Rock Pathway Inhibition

BV2 cells were grown at a density of 1 × 10^5^ cells well^−1^ in 24‐well plates and incubated at 37 °C with 5% CO_2_ for 24 h. Fresh medium and D/P‐g‐PSB and Ibu@D/P‐g‐PSB hydrogels were added and incubated for 24 h. The upper layer of medium or hydrogel was removed and fixed with 4% PFA. Then incubated with 0.1% Triton X‐100 and sealed with 5% BSA. Anti‐ROCK2 (dilution 1:300) was added and incubated at 4 °C overnight. The secondary antibody, Goat Anti‐Rabbit IgG H&L (Alexa Fluor594) (dilution 1:600), was added and incubated at room temperature in the dark for l h. Nuclei were stained with DAPI. The protein expression of the Rock2 was detected by western blotting. The primary antibodies used included: Rock2(ab45171, abcam, the US), Gapdh (10494‐1‐AP, Proteintech, the US). The secondary antibodies used included: HRP‐conjugated Affinipure Goat Anti‐Rabbit IgG(H+L) (SA00001‐2, Proteintech, the US), CoraLite594‐conjugated Goat Anti‐Rabbit IgG(H+L) (SA00013‐4, Proteintech, the US).

### In vitro Cell Experiments—q‐PCR Analysis

BV2 cells (2.8.3) were suspended in TRIzol reagent (Invitrogen, the US), and RNA was extracted using a Total RNA Extraction Kit (Solarbio, China). Purified RNA was quantified using a NanoDrop ND‐1000 (NanoDrop, the US). Subsequently, the mRNA was reverse‐transcribed into cDNA using the PrimeScript RT kit (Takara, Japan). RT‐qPCR was then performed using a LightCycler 960 and TB Green Premix Ex Taq II (Tli RNaseH Plus). The primer sequences used in these experiments are listed in Table S3 (Supporting Information).

### In vitro Cell Experiments—Western Blotting

BV2 cells (2.8.3) were lysed in RIPA lysis buffer (Beyotime, China), and the BCA kit (Solarbio, China) was used for the protein assay. After denaturation with SDS loading buffer (Solarbio, China), each protein suspension was separated into 10% or 6% SDS‐PAGE gels and transferred to 0.22 or 0.45 µm polyvinylidene difluoride membranes (PVDF, Invitrogen, the US). The PVDF membrane was then blocked with 5% BSA for 2 h and incubated overnight at 4 °C with primary antibodies. Then, the secondary antibodies were added for 1 h. Proteins were detected by ultra‐sensitive‐enhanced chemiluminescence using a ChemiDoc MP Imaging System.

### In vitro Cell Experiments—Tunel Staining of Neurons In vitro

Primary neuronal single‐cell suspensions were obtained from the embryonic cerebral cortex and cultured in cell dishes coated with polylysine in DMEM‐HG medium(Gibco, the US). The medium was then replaced with Neurobasal medium with 2% B‐27 supplement (without vitamin A) and 1% penicillin/streptomycin. Primary neurons were fixed in 4% PFA for 30 min. Subsequently, they were treated with 0.3% Triton X‐100 for 5 min. The samples were then incubated with TUNEL's working solution (Beyotime, China) for 60 min at 37 °C in a dark environment. After staining with DAPI for 10 min, the cells were imaged using CLSM.

### In vitro Cell Experiments—Construction of the OGD/R Model

After seven days of culture, primary neurons were used to construct the oxygen‐glucose deprivation/reoxygenation (OGD/R) model. After changing the medium to glucose‐free Duchenne‐modified Eagle's medium (Gibco, the US), the cells were cultured in an anaerobic GENbag culture system under hypoxic conditions for 1 h to achieve OGD. The cells were then cultured in normal serum‐free medium (OGD group) and serum‐free medium with the Ibu@D/P‐g‐PSB hydrogel for 24 h under normal culture conditions.

### In vivo Animal Experiments—Animals

Female C57 mice (6–8 weeks old) were purchased from Beijing Vital River Laboratory Animal Technology Co., Ltd. (Beijing, China). All animal experiments were approved by the Laboratory Animal Ethics and Welfare Committee of the Qilu School of Medicine, Shandong University (Jinan, approval number: 23052).

### In vivo Animal Experiments—Establishment of a Spinal Cord Contusion Model

Because the spinal cord contusion model was similar to a clinical injury, a modified Allen's method was used to establish a rat spinal cord contusion model. Briefly, mice were anesthetized and fixed in the prone position on an experimental table. An incision of ≈2.0 cm in length was made in the direction of the spine, centered on chest 10, and the skin and muscles were incised and separated on both sides. The spinous process was exposed, and the vertebral body was positioned accurately and fully exposed. The thoracic 9–11 vertebrae were removed using bone‐biting forceps to fully expose the thoracic 9–11 spinal cord. A moderate spinal cord contusion model was established by placing an impact rod at the level of spinal cord T10 and free‐falling a 5 g node from a height of 12.5 mm. Blank and drug‐loaded hydrogels were implanted into the injury site, and the untreated spinal cord injury group served as a control. Finally, the muscles and skin were sutured. The hydrogels were injected into the injury area with a volume of 10 µL.

To ascertain the effect of the hydrogels on spinal cord injury repair, adult female mice were randomized into four groups: Sham, SCI, blank @D/P‐g‐PSB hydrogel, and Mel/Ibu@D/P‐g‐PSB hydrogel (*n* = 6). Before the mice resumed voluntary urination, artificially assisted urination was performed using hand pressure on the bladder twice daily.

### In vivo Animal Experiments—Behavioral Functional Recovery Analysis

BMS scores and footprints were used to evaluate spinal cord function. The mice were placed on a platform to move freely, and the BMS score was assessed based on hindlimb walking and limb movement by an observer who was blinded to the grouping. The scores ranged from 0 to 9 points, with 0 indicating no limb movement and 9 indicating normal limb movement. After mice received isoflurane anesthesia, mice motor evoked potentials (MEPs) were measured with an electrophysiological device (BL420N, Tekman, China). Continuous stimulation was performed using a 10 mA current for 1 ms. Automated gait analysis was performed with the CatWalk XT 10.6 platform (Noldus, Netherlands), where ventral plane videography captured paw prints through a glass walkway for subsequent computational evaluation of locomotor patterns. 3D gait parameters, including regularity index, base of support, and stand duration, underwent systematic quantification through the platform's analytical modules.

### In vivo Animal Experiments—Histological Analysis of the Bladder Wall

Mice were deeply euthanized using isoflurane 8 weeks post‐SCI and were perfused with PBS and 4% PFA. The gastrocnemius muscle, bladder, and spinal cord were dissected and separated. After fixation, thoroughly wash with water and replace the moisture in the tissue with a dehydrating agent to facilitate the infiltration of organic solvents. Embed the transparent tissue in a wax block. Cut the paraffin‐embedded tissue into thin slices with a thickness of 6 µm using a slicer. The slices of the bladder wall were used for H&E staining as the following steps. The tissue slices were stained with hematoxylin solution (Solarbio China) for 2 min and the eosin solution (Solarbio China) for 60 s. Then, slices were soaked with ethanol for gradient dehydration (75%, 85%, 95%, 100%) and xylene twice for 1 min each time for transparency. Finally, the slices were covered with neutral gum (Solarbio China). All slices were observed by an optical microscope.

### In vivo Animal Experiments—Immunofluorescence Histochemistry of Spinal Cord and Gastrocnemius Muscle

The slices of the spinal cord and gastrocnemius muscle were paraffin‐embedded sections after fixation with PFA at 4 °C. Subsequently, they were blocked with 5% normal goat serum (Gibco, USA) and 5% BSA mixed with 0.3% Triton X‐100 for 1 h. Primary antibodies were co‐incubated overnight with primary antibody solutions (dilution ratio 1:400). Then, the secondary antibodies were diluted at a ratio of 1:400 and then incubated with the sections for 1 h at 37 °C. Cell nuclei were stained with DAPI, and the samples were visualized using CLSM. The primary antibodies used included: iNOS (22226‐1‐AP, Proteintech, the US), Arg‐1 (66129‐1‐Ig, Proteintech, the US), iba1 (17198S, CST, the US), iba1 (ab283319, abcam, the US), GFAP (ab53554, Abcam, England), Laminin (ab11575, Abcam, England), Tuj1(ab78078, Abcam, England), ChAT (ab178850, Abcam, England), 5HT+ (ab271031, Abcam, England). Tuj1 (ab215037, Abcam, England). The secondary antibodies used included: HRP‐conjugated Affinipure Goat Anti‐Rabbit IgG(H+L) (SA00001‐2, Proteintech, the US), CoraLite594‐conjugated Goat Anti‐Rabbit IgG(H+L) (SA00013‐4, Proteintech, the US). The thickness of gastrocnemius muscle fibers was calculated based on the cross‐sectional area of muscle fibers outlined by Laminin staining. The cross‐sectional area of the muscle fibers in the gastrocnemius muscle of each group of mice was collected at least 10 times (*n* = 10) using Image J in the images captured under the observation field of view.

### In vivo Animal Experiments—In vivo Drug Release

The in vitro drug release of the Mel/Ibu@D/P‐g‐PSB hydrogel was determined by high‐performance liquid chromatography (HPLC). Briefly, prepare a 100 mm DMSO solution of MT and IBU, dilute it with PBS to 1mm to prepare the Mel/Ibu@D/P‐g‐PSB hydrogel (with a solid content of 50%), and then establish a spinal cord contusion model and administer the Mel/Ibu@D/P‐g‐PSB hydrogel treatment to each mouse. Blood samples were taken at regular intervals (1, 2, 3, 4, 5, 6, 7 days) to measure the drug concentration in the serum. The HPLC parameters were as follows: C18 phase column, Acetonitrile – 0.1 mol L^−1^ ammonium acetate (70:30) as the mobile phase, detection wavelength of 265 nm, column temperature of 30 °C, flow rate of 0.8 mL min^−1^, and injection volume of 10 µL.

### In vivo Animal Experiments—In vivo Degardation

The FITC‐labeled gel with a volume of 20 µL was implanted into the back of the mice to investigate biodegradability. Mice were imaged at different time points (0/2/8/14/16/18 days) using a small animal in vivo fluorescence imaging system (IVISSpectrumTM, the US).

### In vivo Animal Experiments—RNA Sequence Analysis

Two weeks after the spinal cord injury, the spinal cord tissue of the injured area was dissected and extracted from the SCI and Mel/Ibu@D/P‐g‐PSB groups. RNA‐sequencing analysis was performed using LC‐Bio Technology. Differentially expressed genes were screened, and Kyoto Encyclopedia of Genes and Genomes (KEGG) and Gene Ontology (GO) analyses were performed accordingly.

### Statistical Analysis

All data were presented as mean ± standard deviation (SD) and were analyzed using Origin 8.0. One‐way ANOVA was used to analyze the differences. Statistical significance was defined as ^*^ *p*<0.05; ^**^ *p*<0.01; ^***^ *p*<0.001; ^****^ *p*<0.0001.

## Conflict of Interest

The authors declare no conflict of interest.

## Supporting information



Supporting Information

## Data Availability

The data that support the findings of this study are available from the corresponding author upon reasonable request.
