# Supplementary material for: Nanogel Integrated Zwitterionic Injectable Hydrogel with Sequential Drug‐Releasing Capability for the Programmable Repair of Spinal Cord Injury
Source: Adv Sci (Weinh). 2025 Nov 12;13(6):e10976. doi: 10.1002/advs.202510976 (PMC12866810; doi:10.1002/advs.202510976)
Supplement: Supplementary file 1 — Supporting Information [file ADVS-13-e10976-s001.docx]

**Nanogel integrated zwitterionic injectable hydrogel with sequential drug-releasing capability for the programmable repair of spinal cord injury**

*Zhijian Wei, Susu Huang, Wencan Zhang, Jiayao Wen, Xiaolong Zhou, Wei He, Xiaoye Yang, Haifeng Wang, Guangxi Zhai, Bin Shi^*^, Lin Jin^*^, Dachuan Wang^*^, Shiqing Feng^*^, and Lei Ye^*^*

**Supporting Information**

**Supporting figures**


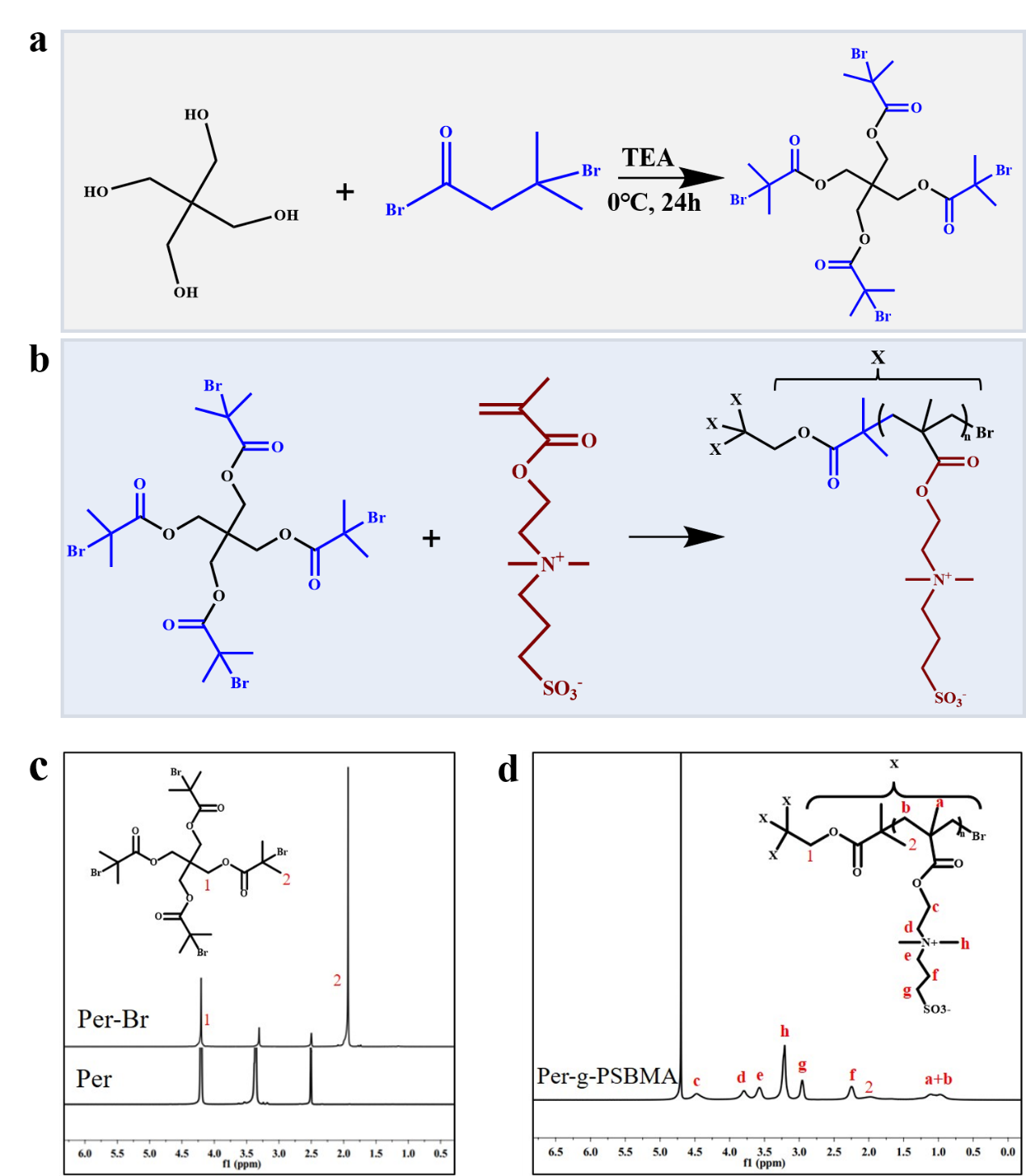


Figure S1: a) Synthesis routes of Per-Br and b) Per-g-PSB; c) ^1^HNMR spectra of Per-Br and d) Per-g-PSB.


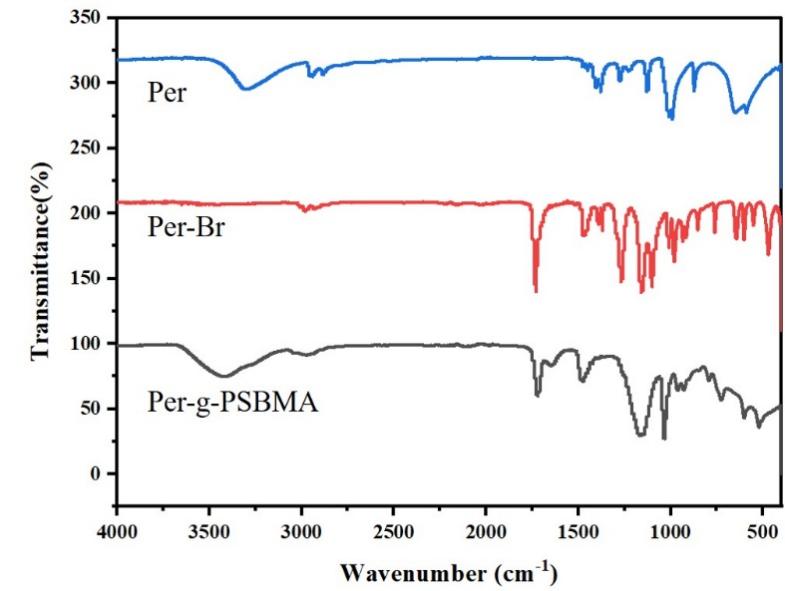


Figure S2: FT-IR spectra of Per, Per-Br and Per-g-PSBMA.


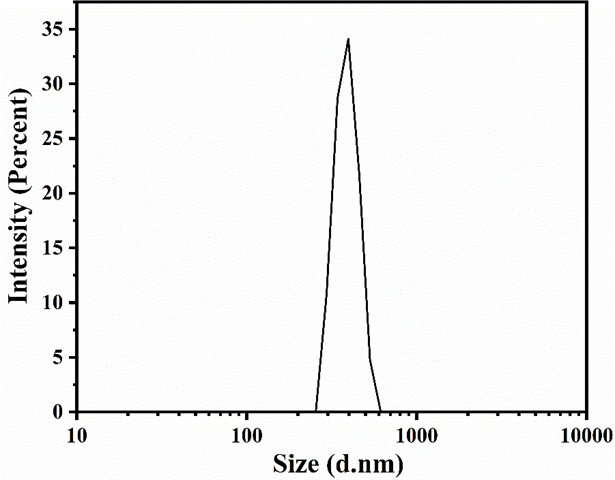


Figure S3. The size distribution of Per-g-PSBMA

Figure S4. The zeta potential of Per-g-PSBMA


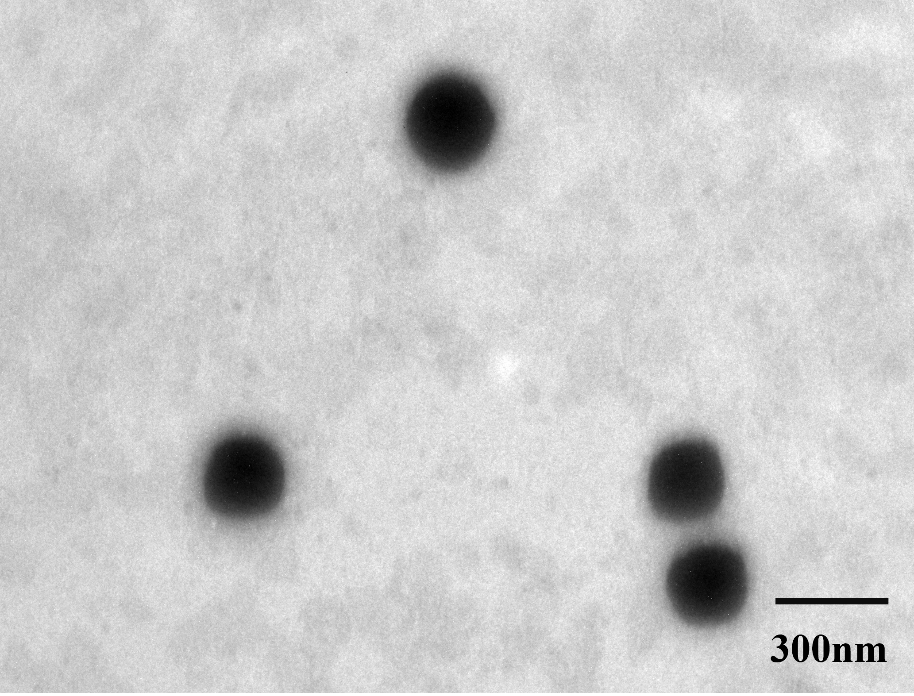


Figure S5. TEM image of Per-g-PSB nanogel (10000×)


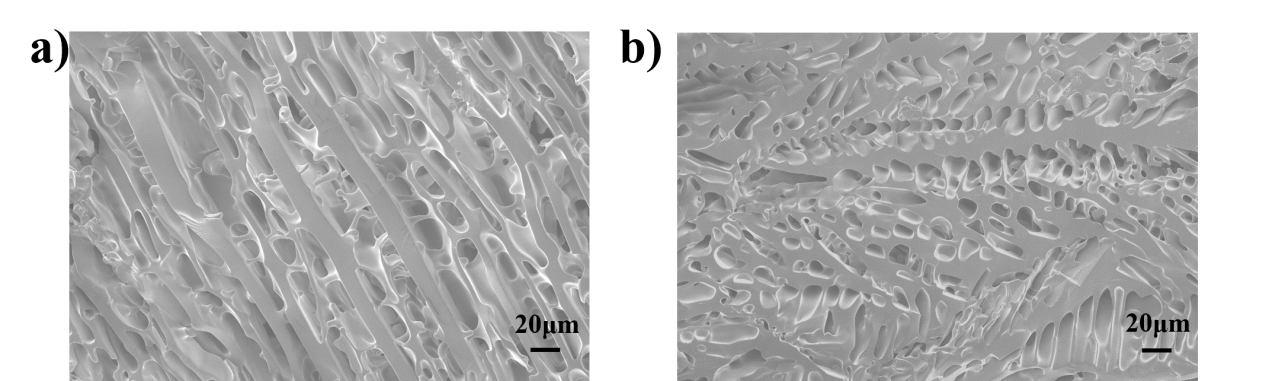


Figure S6: The SEM images of different mass of hydrogels.


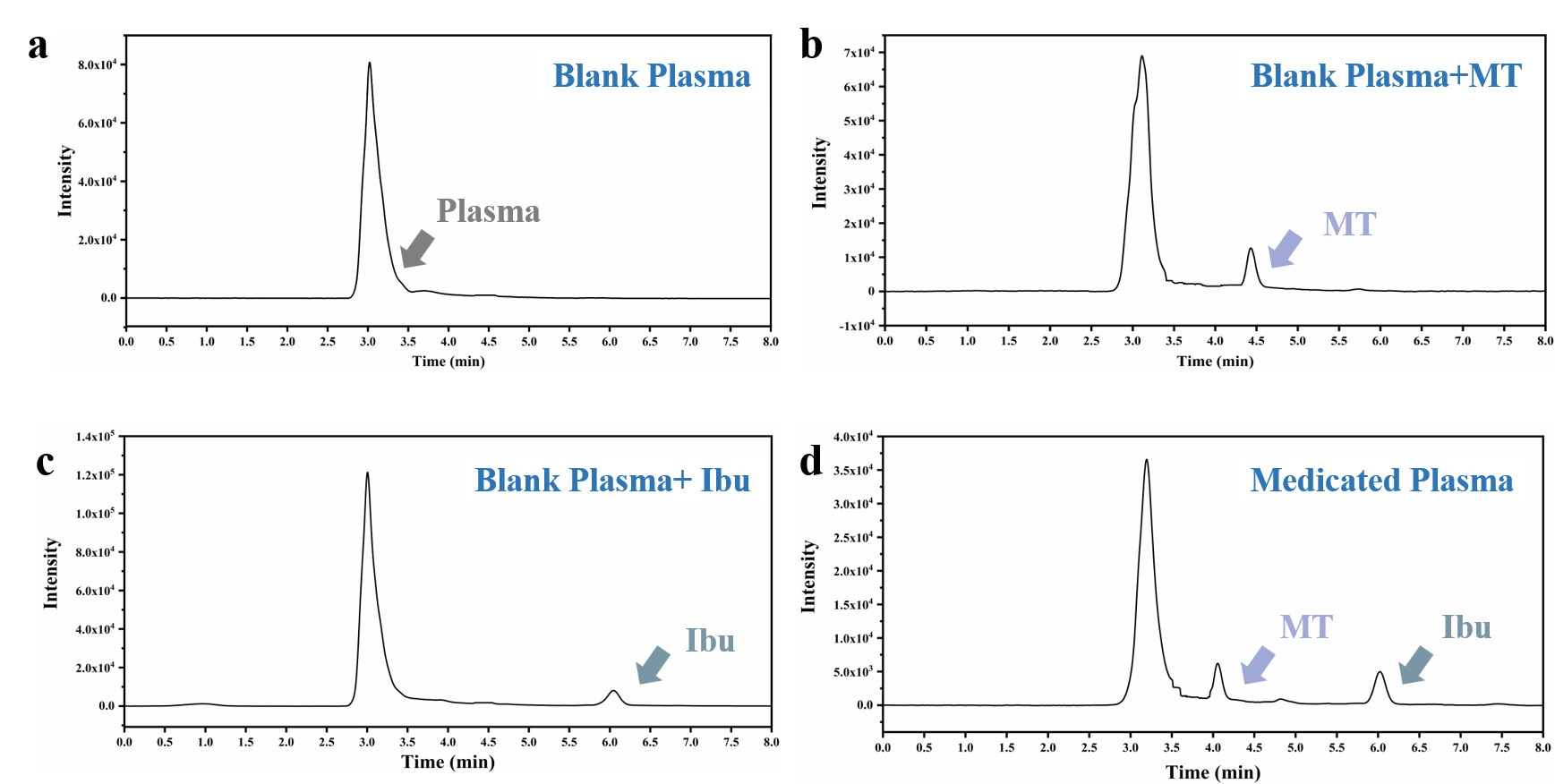


Figure S7: High-performance liquid chromatography spectra of a) Blank plasma, MT-containing plasma, IBU-containing plasma, and Medicated plasma at a detection wavelength of 265 nm.


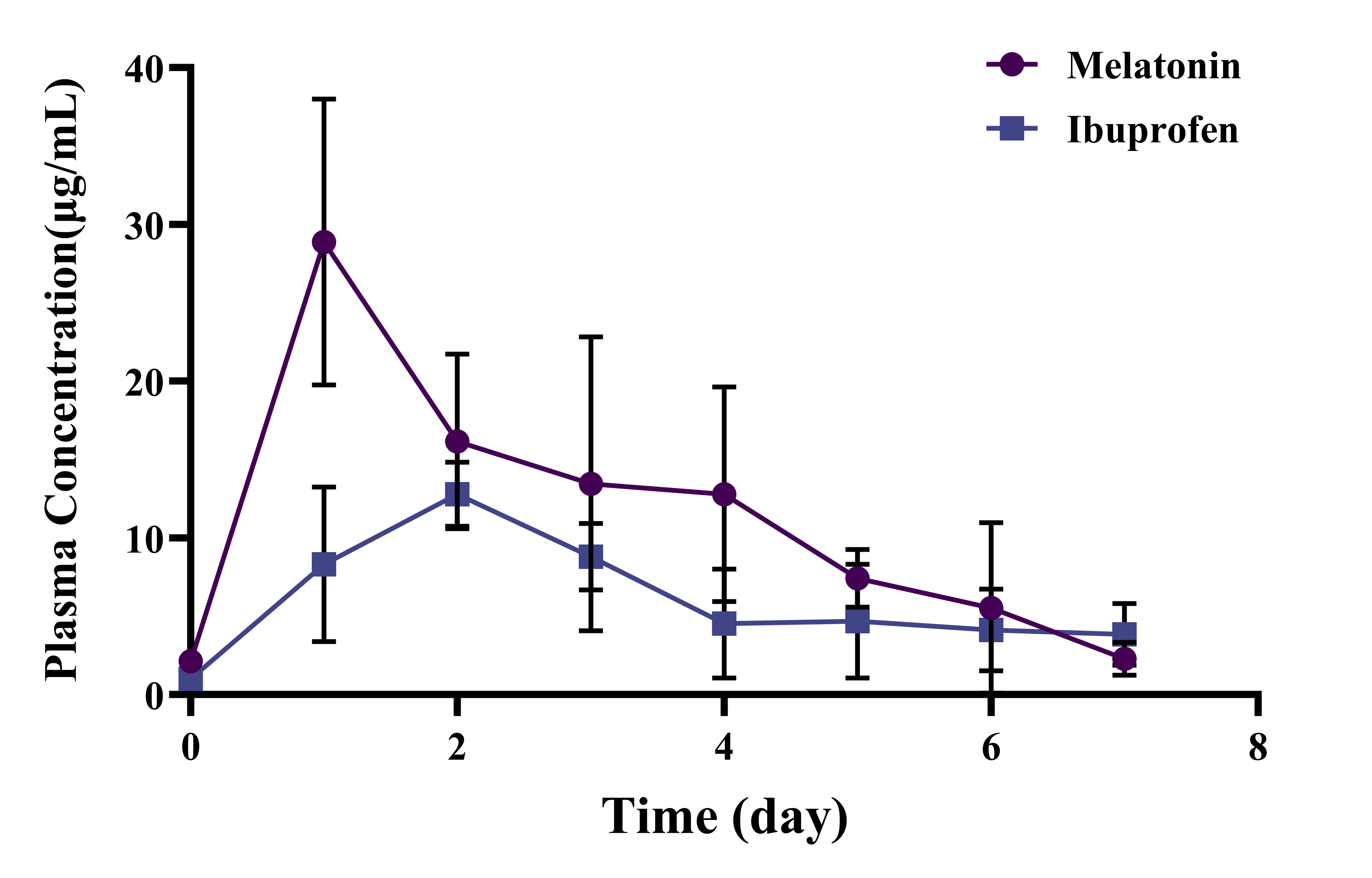


Figure S8: The blood concentration-time curve of ibuprofen and melatonin after administration in mice with spinal cord injury.


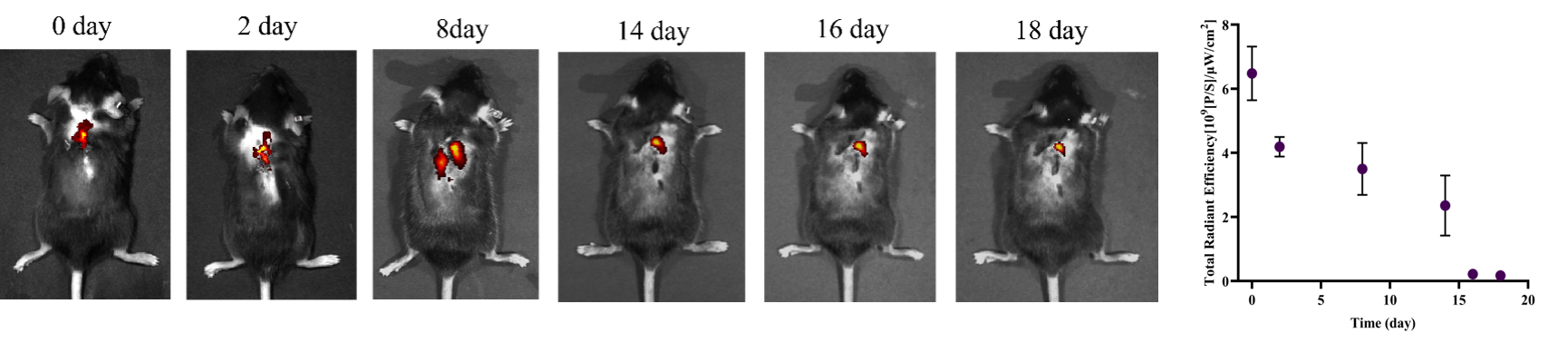


Figure S9: The degradation of D/P-g-PSB in vivo.


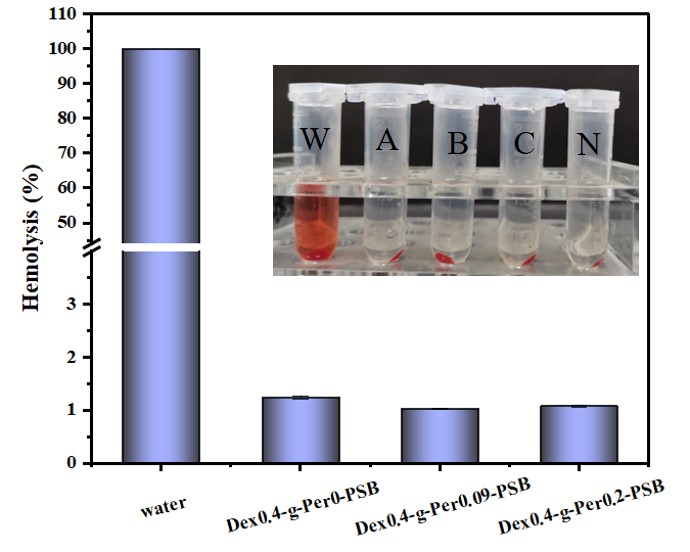


Figure S10. The potential toxicity of the hydrogels. Inset: optical photographs of different sample groups. W: positive; N: negative.


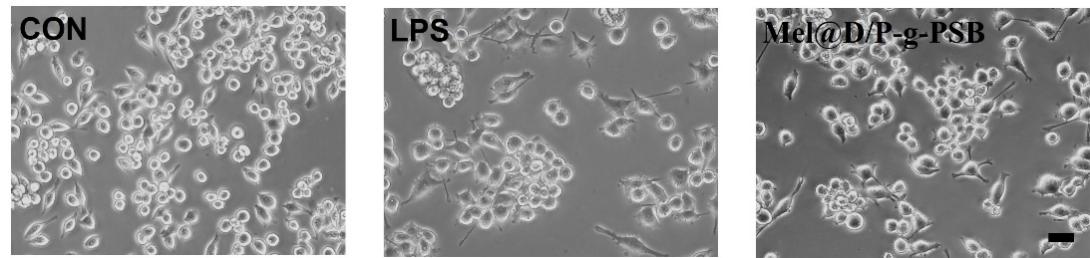


Figure S11: Modeling of LPS inflammation in BV2 cells. Scale bar: 50μm.


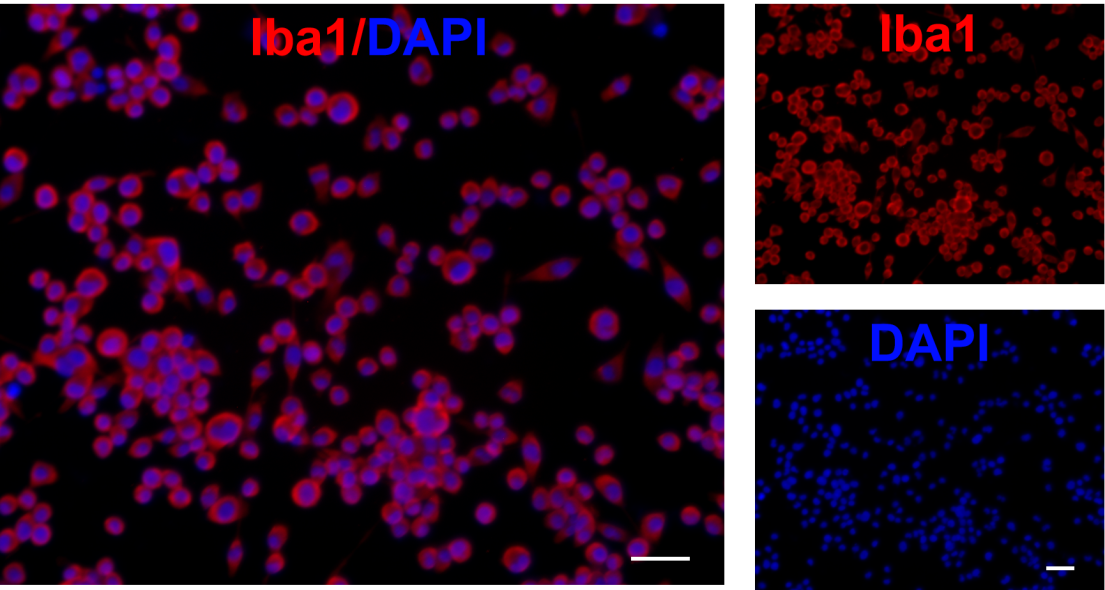


Figure S12: Characterization of Ibal markers for BV2 cells. Scale bar: 50 μm.


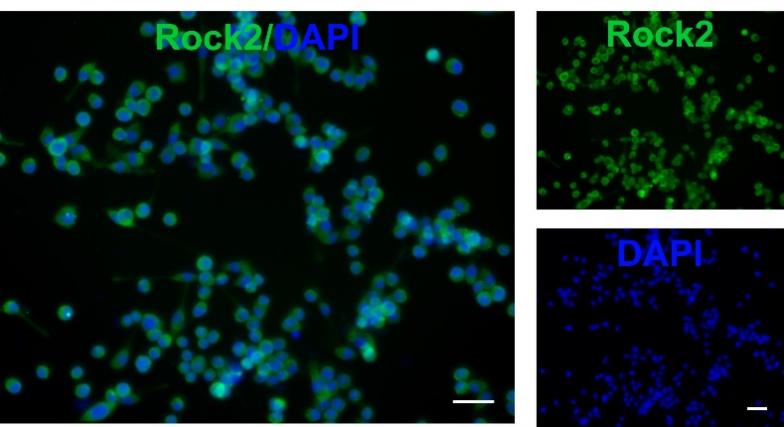


Figure S13: Characterization of ROCK-2 markers for BV2 cells. Scale bar: 50 μm.


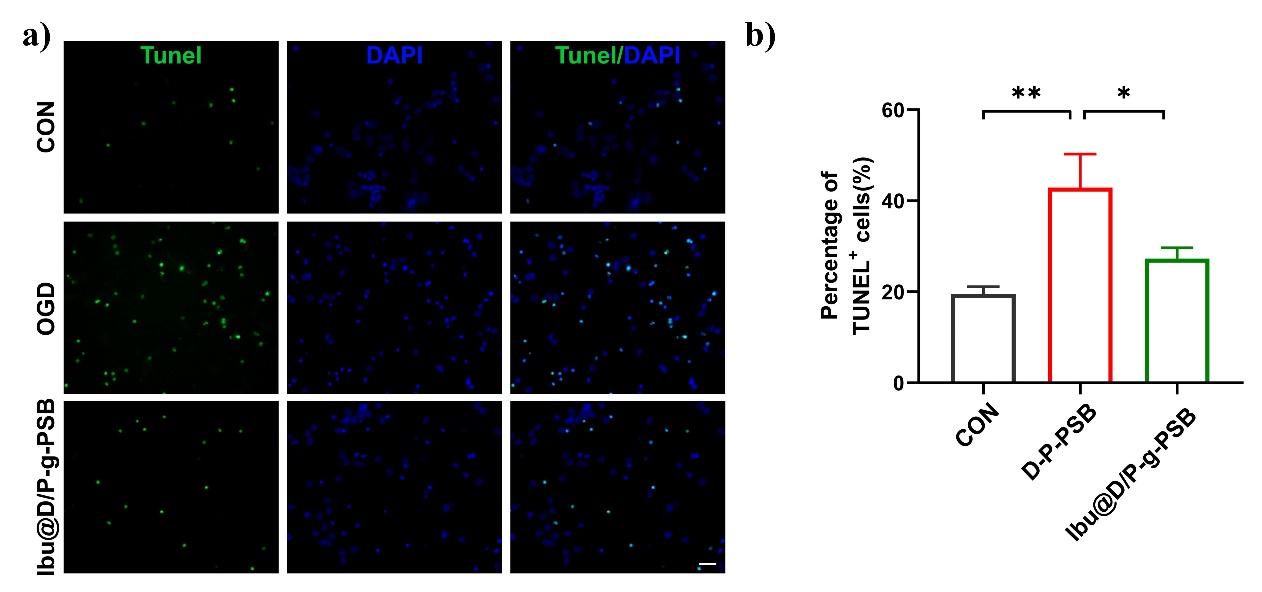


Figure S14: Evaluation of neuronal apoptosis before and after treatment with Ibu@D/P-g-PSB hydrogel. Scale bar: 50 μm.


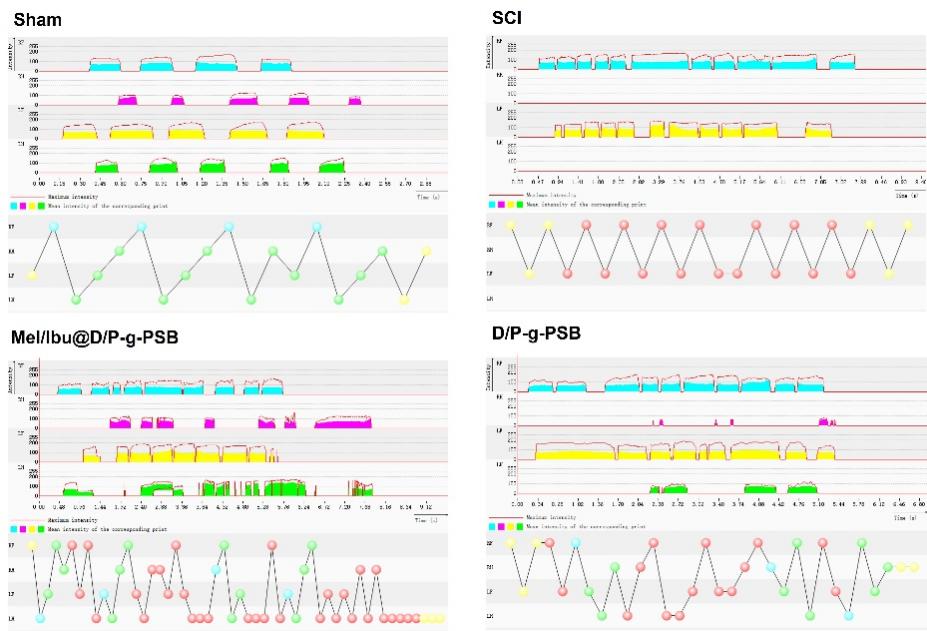


Figure S15: Gait analysis of mice in different groups.


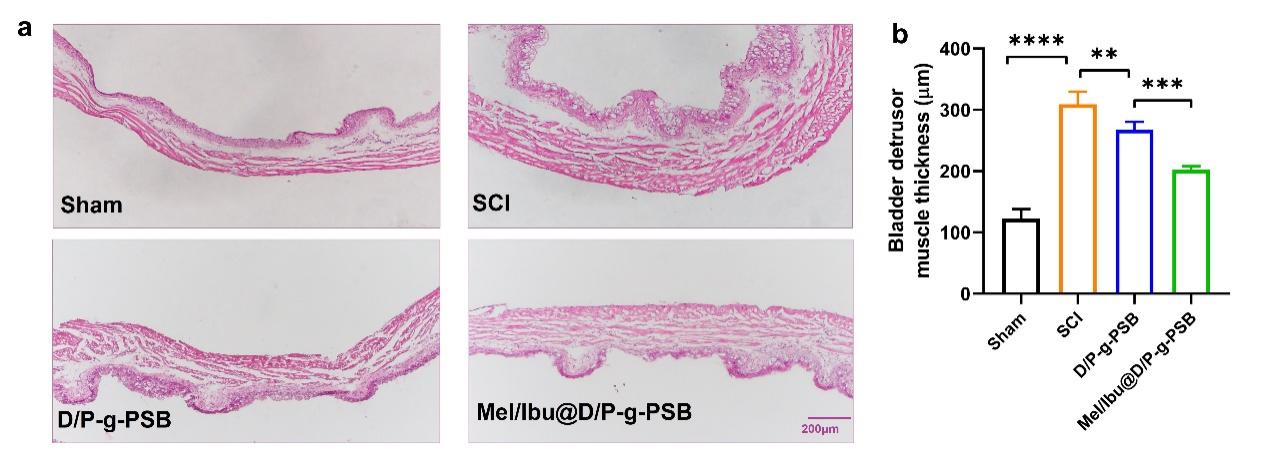


Figure S16: H&E staining of the bladder.


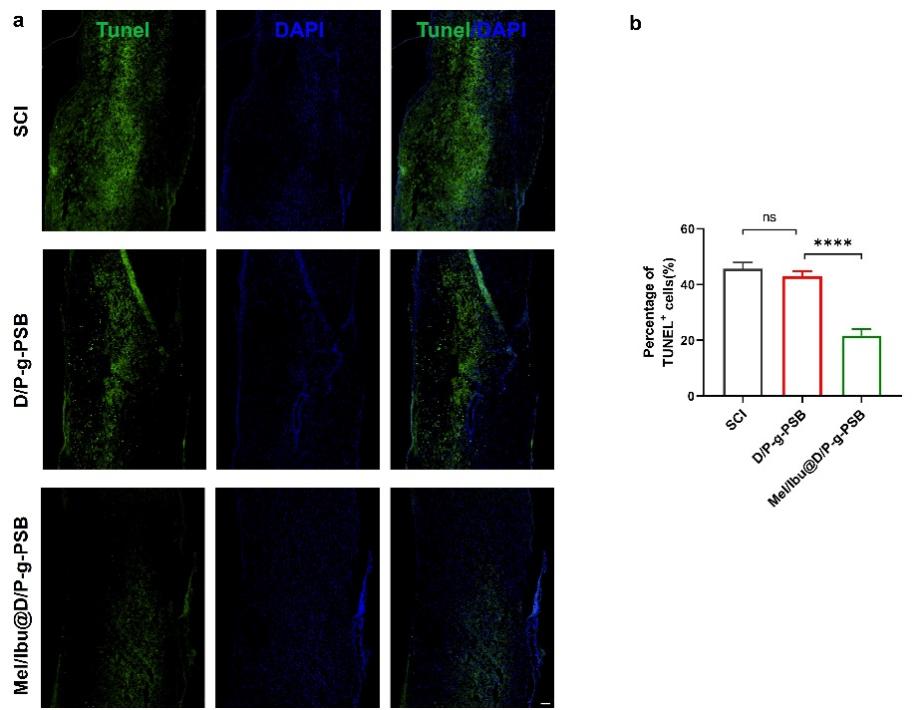


Figure S17: Neuronal apoptosis during the acute phase of spinal cord injury in mice. Scale bar: 200 μm.

Table S1: Yield of Dex-g-PSBMA with different degrees of polymerization.

| Dex-Br | 1 mmol | | | | |
| --- | --- | --- | --- | --- | --- |
| SBMA | 60 mmol | 90 mmol | 120 mmol | **180 mmol** | 240 mmol |
| yield | 73.34% | 90.24% | 77.69% | **81.69%** | 20~50% |

Table S2: Formulation of Dex/Per-g-PSBMA hydrogels.

| Different percentages of the hydrogels | 40% | 45% | 50% |
| --- | --- | --- | --- |
| Dex-g-PSBMA | 0.4 g | 0.4 g | 0.4 g |
| Per-g-PSBMA | 0 g | 0.09 g | 0.2 g |
| 0.9% sodium chloride solution | 0.6 mL | 0.6 mL | 0.6 mL |

Table S3. The primer sequences in the present study.

|  | | |
| --- | --- | --- |
| Geen name | Forward (5' to 3') | Reverse (5' to 3') |
| β-actin | GGCTGTATTCCCCTCCATCG | CCAGTTGGTAACAATGCCATGT |
| iNOS | CCTGCTTTGTGCGAAGTGTC | CCCAAACACCAAGCTCATGC |
| IL-6 | CTTCTTGGGACTGATGCTGGT | CTCTGTGAAGTCTCCTCTCCG |
| TNF-α | AGCCGATGGGTTGTACCTTG | ATAGCAAATCGGCTGACGGT |
| IL-4 | TCACAGCAACGAAGAACACCA | CAGGCATCGAAAAGCCCGAA |
| Arg-1 | TGTCCCTAATGACAGCTCCTT | GCATCCACCCAAATGACACAT |
| CD206 | ACGAGCAGGTGCAGTTTACA | ACATCCCATAAGCCACCTGC |
